# On the Differences in Trimethylaluminum Infiltration into PMMA and PLA Polymers for Sequential Infiltration Synthesis: Insights from Experiments and First-Principles Simulations

**DOI:** 10.1021/acsapm.5c02680

**Published:** 2025-09-15

**Authors:** Michele Perego, Alessia Motta, Karl Rönnby, Forest Tung Jie Yap, Gabriele Seguini, Claudia Wiemer, Michael Nolan

**Affiliations:** † Unit of Agrate Brianza, 9327 CNR-IMM, Via C. Olivetti 2, I-20864 Agrate Brianza, Italy; ‡ Dipartimento di Energia, Politecnico di Milano, Via Ponzio 34/3, 20133 Milano, Italy; § 261183 Tyndall National Institute, University College Cork, Lee Maltings, Cork T12R5CP, Ireland

**Keywords:** PMMA, PLA, sequential infiltration synthesis, vapor phase infiltration, ellipsometry, XPS, density functional theory, swelling

## Abstract

Sequential infiltration synthesis (SIS) is a powerful approach for templated growth of solid materials, such as oxides or metals, that exploits the difference in interaction of a precursor molecule with a polymer or block copolymer. While there have been studies showing that infiltration of trimethyl-aluminum (TMA) in polymers can be used to grow Al_2_O_3_, there are still many atomic level details of the SIS process that require more investigation, including the origin of the differences in infiltration of TMA into different polymers. In this paper, we investigated in detail the infiltration of Al_2_O_3_ into poly­(methyl methacrylate) (PMMA) and poly­(lactic acid) (PLA) experimentally and theoretically. SIS was performed in a standard ALD reactor, operating at 70 °C in quasi-static mode, using TMA and water as the metal and oxygen precursors, respectively. Operando spectroscopic ellipsometry and ex-situ X-ray photoelectron spectroscopy (XPS) evidenced that Al_2_O_3_ incorporation in PLA is significantly higher than in PMMA even if, in both cases, TMA incorporation occurs through the formation of an Al–O covalent bond at the C–O–C group. The extent of swelling of the polymers upon TMA infiltration is assessed and is clearly larger for TMA in PLA than in PMMA. First-principles density functional theory (DFT) calculations highlighted that both polymers display swelling upon TMA infiltration, saturating with increasing TMA, consistent with operando ellipsometry observations. The DFT results also show the origin of the larger swelling in PLA compared to TMA. Changes in vibrational modes of carbonyl backbone groups in the polymers are used to demonstrate TMA-polymer interactions from both experiment and simulation. The differences in TMA infiltration and swelling arise from differences in the TMA-polymer C–O–C group interaction, which is more exothermic in PLA than in PMMA, in agreement with experimental results. The combination of experimental and theoretical studies herein reported provides a toolkit to disclose the complexities of SIS at the molecular level.

## Graphical abstract

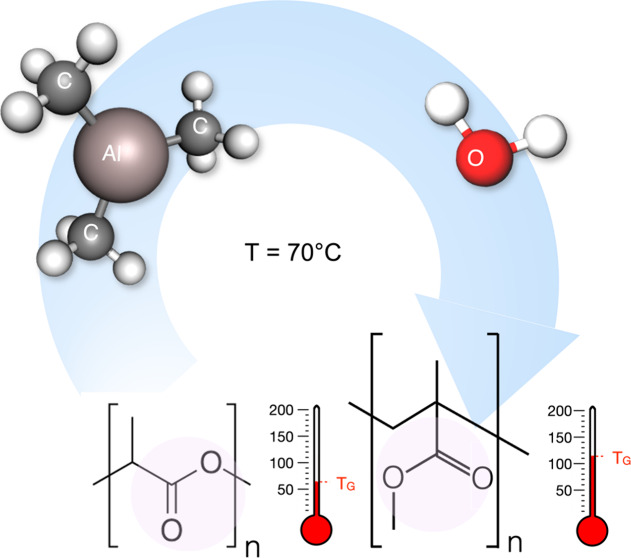

## Introduction

Sequential infiltration synthesis (SIS) is a vapor phase infiltration (VPI) technique to produce organic–inorganic hybrid materials and/or inorganic nanostructures through infiltration of an inorganic precursor into a suitable polymer template.^1–5^ SIS uses the same approach as atomic layer deposition (ALD) through introducing precursors/coreactants in separate, sequential steps and exploiting self-limiting precursor reaction chemistry to deposit target materials.^6^ Despite the broad interest in and importance of SIS for semiconductor nanolithography,^7–9^ nanopatterning,^10–12^ organic electronics,^13–15^ and water membrane technology,^16–19^ the number of inorganic materials that can be grown by SIS remains quite limited.^20–23^ The potential for expanding the scope of SIS is significant, particularly by drawing from the extensive body of ALD chemistry and employing a wider range of polymer–precursor combinations. Accordingly, to expand the library of materials that can be synthesized using this technique, widening its application fields and appeal, further experimental and theoretical studies are necessary to better elucidate the kinetics of sorption, diffusion, and reaction of precursors in the different polymer matrices. In this respect, the understanding of the fundamental physicochemical mechanisms governing the infiltration of SIS precursors and coreactants into the polymer matrix is very important to help with developing modeling tools for SIS, similar to the state of modeling of ALD,^24,25^ with predictive capabilities, that will support the community in the search for novel precursors and the establishment of proper infiltration protocols.

Infiltration of Al_2_O_3_ into poly­(methyl methacrylate) (PMMA) thin films using trimethyl aluminum (TMA) and H_2_O as precursors has been widely investigated and is commonly considered as a reference system in the scientific community. Variation of sorption, diffusion, and reaction of TMA into this specific polymer as a function of processing parameters have been well studied.^26–30^ In particular, upon increasing the processing temperature, a progressive enhancement of TMA diffusivity is determined,^24,28^ but a concomitant reduction of TMA solubility and reactivity is induced, significantly decreasing the amount of TMA trapped into the polymer matrix.^28,31,32^ As a consequence, the effective incorporation of Al_2_O_3_ into the PMMA matrix is generally limited, irrespective of the processing temperature. Recent results have highlighted that several polymers, like poly­(ethylene terephthalate glycol) (PET-G),^32^ polycaprolactone (PCL),^33^ poly­(lactic acid) (PLA), and poly­(butylene succinate) (PBS), exhibit significantly higher reactivity to TMA compared to PMMA.^34^ In particular, by monitoring the evolution of the film thickness during the infiltration process, *Petit et al.* demonstrated extremely efficient infiltration of Al_2_O_3_ into PET-G arising from the irreversible reaction of TMA with the polymer matrix which limits the out-diffusion of TMA during the purging step.^32^ Biswas et al. studied infiltration of Al_2_O_3_ into PMMA and PCL, demonstrating that the interaction of PCL with metal precursors is very strong, even though its carbonyl (C=O) and ester (C–O–R) functional groups are similar to those of the more weakly interacting PMMA.^33^ Similarly, Padbury et al. reported that nearly 100% of the reactive groups in the PCL matrix react with TMA molecules, leading to a significant amount of Al_2_O_3_ incorporated into the polymer matrix during a single SIS cycle.^35^ Finally, Motta et al. reported homogeneous growth of Al_2_O_3_ throughout the entire thickness of a 30 μm thick PBS freestanding membrane, indicating an extremely high diffusivity of TMA into this polymer matrix.^34^ Moreover, Al_2_O_3_ mass uptake in PBS thin films was demonstrated to be much higher than that in standard PMMA films, under the same process conditions. Consequently, having the same functional groups does not guarantee strong infiltration of the inorganic phase into the polymer matrix,^36,37^ but more understanding of the details of precursor infiltration is required.

Overall, data in the literature suggest that the limited reactivity of TMA with the PMMA matrix is the exception rather than the rule. Interestingly, all these polymers are characterized by the presence of ester groups that are expected to act as reactive sites for TMA during the SIS process, but the works discussed above demonstrated that the presence of ester groups alone in the polymer is not sufficient to drive the SIS chemistry, and other factors will also be important. The different position of the reactive sites along the polymer chains appears to play a crucial role, resulting in a clear variation of Al_2_O_3_ incorporation into the different polymer matrices.^32–35,38^ More information about the reaction of TMA with these functional groups is necessary to better understand polymer–precursor interactions and investigate the fundamental mechanisms governing inorganic phase growth into a polymer matrix during the SIS process. However, this is challenging to elucidate with only experimental techniques, and first-principles simulations can contribute significantly to growing our understanding of SIS chemistries.

In this work, the infiltration of Al_2_O_3_ into PMMA and PLA thin films was investigated experimentally and, for the first time, theoretically with first-principles density functional theory (DFT) simulations using both nonperiodic and periodic models of the TMA-polymer interactions. Infiltration processes were performed in a conventional ALD reactor, operating in quasi-static mode at 70 °C, using TMA and H_2_O as metal and oxygen precursors, respectively. Information about solubility and reactivity of TMA molecules in the two polymer matrices was obtained by monitoring polymer swelling by operando spectroscopic ellipsometry (SE) and analyzing chemical composition of the infiltrated polymers by ex-situ X-ray photoelectron spectroscopy (XPS). Experimental results were combined with DFT simulations of explicit TMA infiltration and polymer swelling to provide a consistent picture of TMA interaction with the different polymer matrices, identifying the energetically favorable binding configurations of TMA with PMMA and PLA polymer chains and elucidating the origin of the differences between the two polymers. In addition, we demonstrated that polymer swelling can be well described and, hence, predicted within DFT simulations, which open new avenues to predicting novel precursor-polymer systems for SIS applications.

## Methods

### Sample Preparation

1 × 1 cm^2^ Si samples were cut from a n-type (100) Si wafer. Si substrates were cleaned with 2-propanol and acetone in an ultrasonic bath and subsequently dried by using a stream of ultrapure N_2_. A 30 μm thick free-standing PLA film was supplied by Corapack. PLA was dissolved in chloroform, and the starting material was characterized by size exclusion chromatography (SEC) measurements and differential scanning calorimetry (DSC) analysis. The graphs showing the SEC and DSC data are reported in the Supporting Information (Figures S1 and S2). PLA has molecular weight *M*_n_ = 151 kg/mol and polydispersity PDI = 1.24. The glass transition temperature of PLA was determined to be *T*_G_ ∼ 57 °C. The starting PLA material after dissolution in chloroform and subsequent solvent evaporation was found to be amorphous. A clear evidence of crystallization (*T* > 110 °C) and melting at (*T* > 150 °C) was detected when annealing the polymer, suggesting that the PLA polymer is stereoregular. The PLA containing solution was spin coated on the 1 × 1 cm^2^ Si samples forming ∼15 nm thick polymer films. These polymeric films are amorphous, and no crystallization is expected to occur during processing at 70 °C. PMMA (*M*_n_ = 15 kg mol^–1^, PDI = 1.09) was acquired from Polymer Source. Inc. and dissolved in toluene. Then ∼15 nm thick PMMA films were prepared by spin coating the PMMA solution on the 1 × 1 cm^2^ Si samples. Before infiltration, the PMMA films were annealed at 200 °C for 300 s on a hot plate to remove residual toluene. The chemical structures of PMMA and PLA are reported in Figure 1a together with the indication of the corresponding glass transition temperatures.

**Figure 1:**
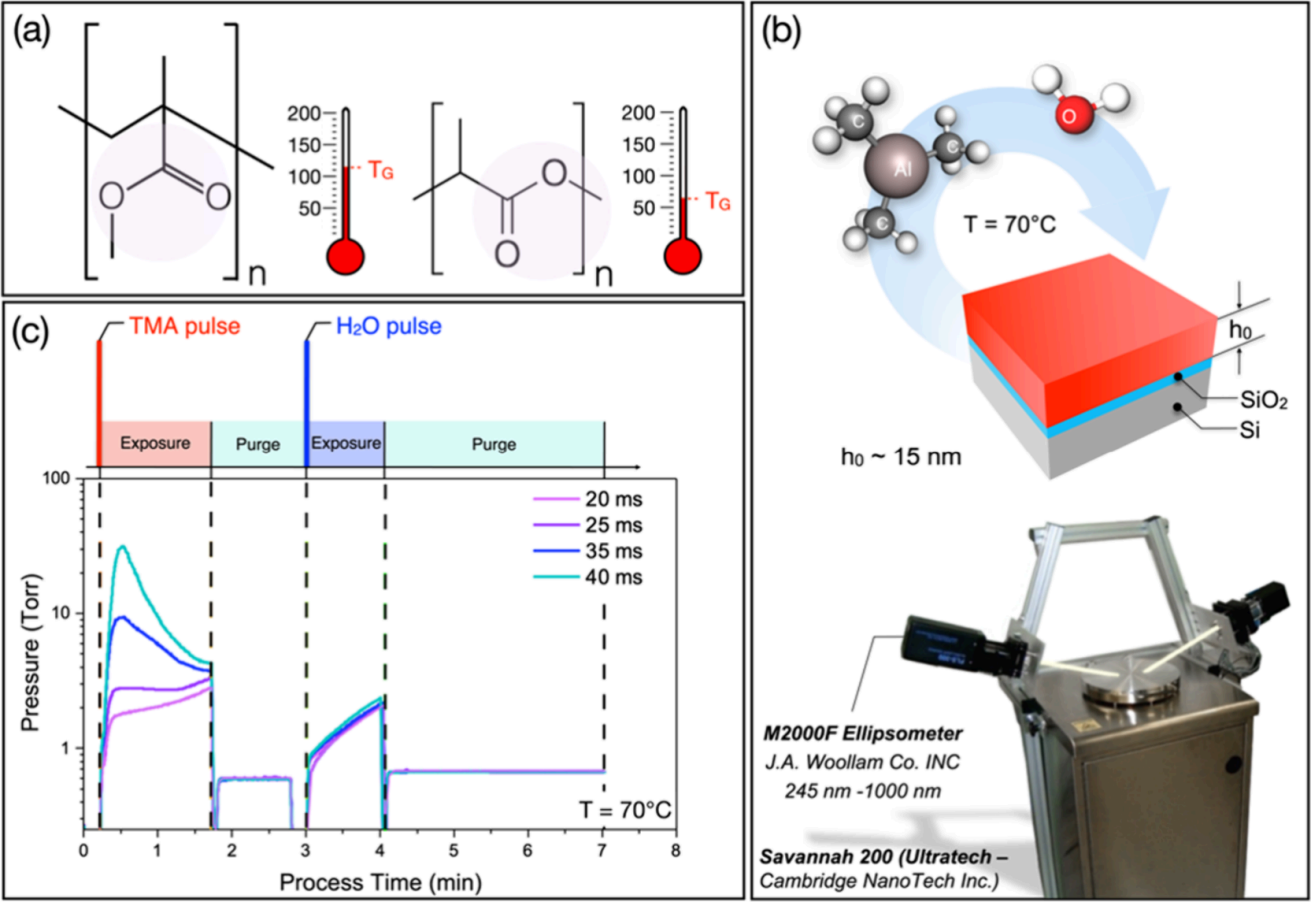
(a) Chemical structure of PMMA and PLA molecules with an indication of their glass transition temperatures. The pink shadows highlight the position of the ester groups in the two molecules. (b) Scheme of the experimental setup showing the ALD reactor equipped with the in situ ellipsometer and the structure of the infiltrated samples. (c) Scheme of the typical SIS cycle along with the pressure evolution in the growth chamber for processes characterized by different durations of the TMA pulse. The duration of the H_2_O pulse was kept fixed (*t* = 15 ms) in all the processes.

### Sequential Infiltration Synthesis Process

SIS processes were performed at 70 °C in a commercial cross-flow ALD reactor (Savannah S200, Ultratech Cambridge NanoTech) utilizing TMA and H_2_O as organometallic and oxygen precursors, respectively, as shown in Figure 1b. This processing temperature is expected to promote Al_2_O_3_ incorporation in the PMMA film.^31,32,34^ Before starting the process, the samples were kept at 70 °C for 30 min under N_2_ flow (100 sccm, 0.66 Torr) to guarantee proper thermalization of the growth chamber and desorption of water molecules trapped in the polymer films during exposure to air. No significant variation of PLA and PMMA film thickness was observed upon thermalization. The SIS cycle comprised the following steps: TMA pulse, TMA exposure, TMA purge, H_2_O pulse, H_2_O exposure, and H_2_O purge. The duration (*t*_TMA_) of the TMA pulses was varied from 25 to 40 ms, while the duration of the H_2_O pulse was 15 ms. The chamber was kept in a static vacuum during the 90 s long TMA and 60 s long H_2_O exposure steps by closing inlet and outlet valves. TMA and H_2_O purge steps were performed by flowing N_2_ into the growth chamber to guarantee complete removal of unreacted precursor molecules and reaction byproducts. TMA purge time was 60 s. H_2_O purge time was 180 s. A scheme of the SIS cycle and the pressure evolution into the ALD reactor is reported in Figure 1c. Experimental data about pressure in the growth chamber were automatically recorded by the system during the infiltration process using a Pirani gauge. A change of the *t*_TMA_ value determines a variation of the chamber pressure, consistent with the idea of a modification of the TMA partial pressure in the growth chamber. After the SIS process, the samples were exposed to an O_2_ plasma (40 W, 10 min) to remove the polymer, resulting in the formation of residual Al_2_O_3_ films on the Si substrate.

### Polymer Characterization

SEC analysis was performed with a 590 Waters chromatograph equipped with Waters HSPgel HR3 and HR4 columns and a refractive index detector. The analysis was carried out at 25 °C using THF as the solvent at a flow rate of 0.3 mL/min. The calibration curve was obtained using polystyrene standards with molecular weight ranging from 1000 to 100,000 g/mol. DSC analysis was carried out by a Mettler-Toledo Calorimetry model 821e instrument on approximately 5 mg of polymer placed in alumina crucibles. Heating and cooling ramps of 10 °C/min were performed between −50 and 180 °C.

### Spectroscopic Ellipsometry

SE data were collected by using a M-2000F (J. A. Woollam Co. Inc.) rotating compensator ellipsometer equipped with an Xe lamp. To enable operando measurements, the ALD reactor was equipped with a modified lid with two quartz windows that allow the incident light to reach the sample and the reflected light to be detected at a 70° angle with respect to the normal of the substrate plane. The assembly of the spectroscopic ellipsometer on top of the ALD reactor is shown in Figure 1b. Ex-situ SE measurements were performed at a fixed 75° incidence angle. The SE data were collected across a wavelength range spanning 250–1000 nm with an acquisition time of 1.6 s. The data were analyzed using version 2.3 of the EASE software package (J.A. Woollam Co. Inc.). The thickness and refractive index of the polymer films during and after the SIS process were determined using a film stack model composed by a polymer layer on top of the thin SiO_2_ film naturally formed on the surface of the Si substrate. A Cauchy layer model was used for the polymer layer. For each sample, the thickness of the native SiO_2_ layer on top of the Si substrate was measured before spin-casting and kept fixed during the analysis of the SE data following a procedure that is described in details in previous publications.^27–29^

### X-ray Photoemission Spectroscopy

XPS measurements were performed using a PHI 5600 instrument equipped with a monochromatic Al Kα X-ray source (1486.6 eV) and a concentric hemispherical analyzer. The spectra were collected at a takeoff angle of 45°. Low-resolution spectra were acquired with a pass energy of 93.9 eV. High-resolution spectra were acquired with a pass energy of 11.75 eV. Calibration of the spectrometer was accomplished by using polycrystalline gold, silver, and copper samples. The binding energies of the Au *4f*_*7/2*_, the Ag *3d*_*5/2*_, and the Cu *2p*_*3/2*_ core lines were determined to be 84.0, 368.3, and 932.7 eV, respectively. The analysis of the XPS spectra was performed by using Winspec software. To correct the energy shift induced by charging of the polymer films during the XPS measurements, the C 1*s* core level originated by the carbonyl group was used as a reference signal. The binding energy of this C *1s* signal was set to 289 eV.^39–41^ The high-resolution spectra were fitted by using Shirley background and Voight functions to identify the different core level signals.

### Density Functional Theory Simulations

Two model systems are used for Density Functional Theory simulations of complementary aspects of TMA infiltration in PLA and PMMA. The first model, similar to that previously used for RuO_4_ infiltration in polystyrene and PMMA,^42^ is a nonperiodic, gas-phase model that uses an oligomer of ten monomers for each polymer and standard DFT relaxations. The nonperiodic DFT calculations were performed with the TURBOMOLE software using the PBE0 hybrid exchange-correlation functional with a split valence, def-SV­(P), basis set, which provides reliable results for similar calculations.^43–45^ The self-consistent field (SCF) convergence criterion was 10^–6^ Ha, and the structures were relaxed until an energy convergence of 10^–3^ Ha was reached. IR vibrational modes were calculated by using analytical force constants within the harmonic oscillator approximation.

The second model is a periodic polymer supercell with two eight monomer chains of each polymer. This allows us to explore the impact of TMA content in the polymer, in particular the TMA incorporation and swelling of the two polymers, facilitating for the first time a direct comparison with the experimental results, and allows us to assess limitations associated with a nonperiodic oligomer model of the polymer. Two further periodic models are used: the large model is used for infiltration and swelling described above and a smaller model uses a single six monomer chain for the more computationally expensive calculation of the IR vibrational modes upon TMA infiltration and adduct formation.

The periodic DFT calculations were carried out using the Vienna Ab initio Simulation Package (VASP) 5.4.^46–48^ The Perdew–Burke–Ernzerhof (PBE) generalized gradient approximation of the exchange-correlation functional was used.^49^ An 800 eV plane-wave cutoff energy was used, which minimizes the Pulay stresses during ion and lattice relaxations. Projector augmented wave (PAW) potentials were used, with 1 valence electron for hydrogen, 4 for carbon, 6 for oxygen, and 3 for aluminum.^50^ Gaussian smearing with a width of 0.1 eV was used for all of the calculations. Grimme’s version 3 dispersion correction was included to improve the description of noncovalent interactions between the polymer chains.^51^ Structural relaxions used an electronic convergence criterion of 10^–4^ eV for each ionic step and a force convergence of 0.02 eV/Å. Phonon vibration modes were calculated using a finite central difference with a displacement of 0.015 Å, using an electronic convergence criterion of 10^–6^ eV.

## Experimental Results

### Spectroscopic Ellipsometry

Operando SE measurements allow the real-time monitoring of the thickness evolution of the polymer matrix during the different steps of the SIS process.^27,52^ Accordingly, the swelling ε­(*t*) of the polymer template during the SIS process can be determined using the following equation:

$$\varepsilon (t)=\frac{h(t)-h_{0}}{h_{0}}$$ 1

where *h*(*t*) indicates the thickness of the PMMA film at process time *t* and *h*_0_ the thickness of the PMMA film at the beginning of the process, i.e., at process time *t* = 0. The curves depicting the swelling evolution as a function of time for the 15 nm thick PLA and PMMA films during a single SIS cycle at *T* = 70 °C are reported in Figure 2a,b, respectively. The vertical black dashed lines mark the boundaries among the different steps of the SIS process. For each polymer matrix, different swelling curves were obtained by changing the duration (*t*_TMA_) of the TMA pulse from 20 to 40 ms. All of the samples exhibit similar swelling curves characterized by a fast swelling during the initial stages of the TMA exposure step followed by a level-off to a maximum swelling value (ε_MAX_). This evolution is consistent with the idea that TMA molecules uniformly permeate the 15 nm thick polymer layers, achieving a saturation condition with a concentration of TMA into the polymer matrix that depends on the solubility of TMA into the specific polymer under investigation.

**Figure 2:**
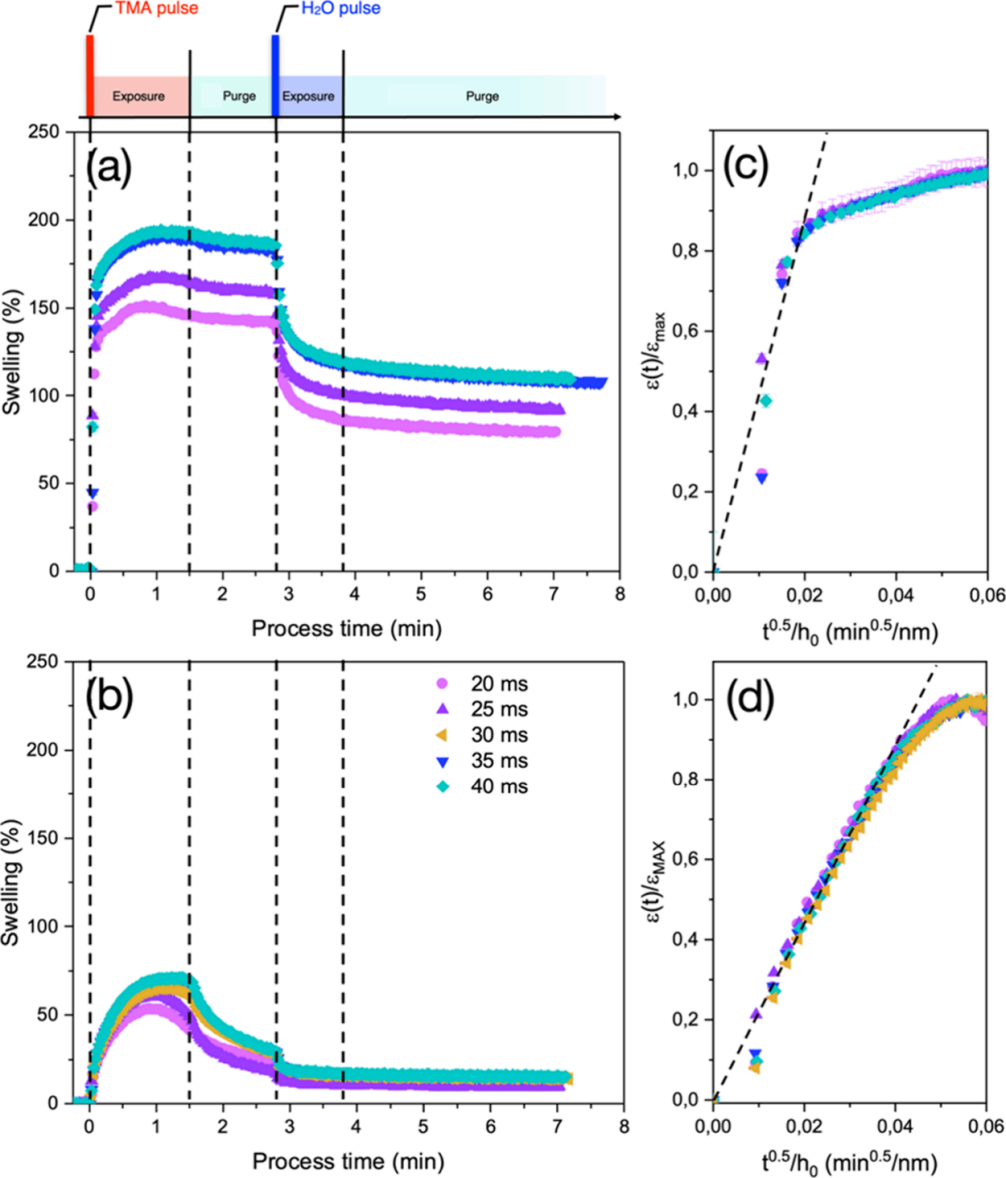
Swelling evolution as a function of time for 15 nm thick PLA (a) and PMMA (b) thin films deposited on silicon substrates during the different steps of the sequential infiltration process. Black dashed lines mark the boundaries among the different steps of the process which is schematically depicted on top of graphs. The different curves correspond to different durations of the TMA pulse that is performed to inject the metal precursor into the growth chamber. Normalized swelling evolution as a function of *t* ^1/2^/*h*_0_ for 15 nm thick PLA (c) and PMMA (d) thin films. The black dashed lines correspond to the fitting of the experimental data with eq 2.

The swelling of the PLA matrix is much faster than that of PMMA, with a much steeper swelling variation during the initial stages of the TMA exposure step, indicating that the diffusivity of TMA is higher in PLA than in PMMA. This is clearly highlighted in Figure 2c,d showing the evolution of the normalized swelling ε­(*t*)/ε_MAX_ as a function of *t* ^1/2^ */h*_0_ for PLA and PMMA thin films, respectively. Previous studies^27,28,52^ demonstrated that TMA diffusion into PMMA is governed by Fickian diffusion during the initial stages of the TMA exposure step, with a progressive increase of the ε­(*t*)/ε_max_ that is perfectly described by the following equation:

$$\varepsilon (t)/\varepsilon_{\max}∼4\sqrt{D/\pi }t^{1/2}/h_{0}$$ 2

In this Fickian diffusion regime, the diffusion coefficient is simply determined as the slope of the linear fit (black dashed lines) of the ε­(*t*)/ε_max_ curves as a function of *t* ^1/2^/*h*_0_ using eq 2. In this way, the diffusion coefficient of TMA in PMMA at 70 °C is determined to be 1.8 ± 0.1 × 10^–14^ cm^2^/s, in perfect agreement with previous results in the literature.^27,28,52^ In the case of PLA, the fitting of the ε­(*t*)/ε_max_ curves with eq 2 is somehow questionable because the polymer films exhibit a more complex swelling evolution than PMMA. In particular, the initial swelling for PLA films is extremely fast, making it difficult to identify a region with a pure linear evolution of the ε­(*t*)/ε_max_ curves. Accordingly, the reliability of the fitting procedure is limited, and the diffusivity value, which is found to be ∼4.9 ± 0.3 × 10^–13^ cm^2^/s, has to be considered only as a rough indication of the effective TMA diffusion coefficient into the PLA matrix.

According to Henry’s law, the higher the TMA partial pressure in the growth chamber, the higher the TMA concentration in the polymer matrix and consequently its swelling. In this respect, both polymers exhibit a similar trend: a progressive increase of ε_MAX_ was induced when increasing *t*_TMA_ from 20 to 35 ms, while no further ε_MAX_ increase was observed for *t*_TMA_ = 40 ms, as shown in Figure 3a,b for PLA and PMMA, respectively. These results suggest that the solubility limit of TMA in these polymer templates was achieved. It is worth noting that the ε_MAX_ values in the case of the PLA samples are significantly larger than the ones obtained in the case of the PMMA samples consistently with the idea that a larger amount of TMA has been incorporated into the PLA samples. In this respect, we remind that the SIS process was performed at *T* = 70 °C: this temperature is slightly above *T*_G_ of PLA and significantly below *T*_G_ of PMMA. Consequently, during the infiltration experiments, PMMA is in the glassy state, while the PLA is expected to be in the rubbery state. In principle, the larger free volume and chain mobility in the rubbery PLA film could partially account for the significant difference in swelling of the two polymers during TMA exposure.

**Figure 3:**
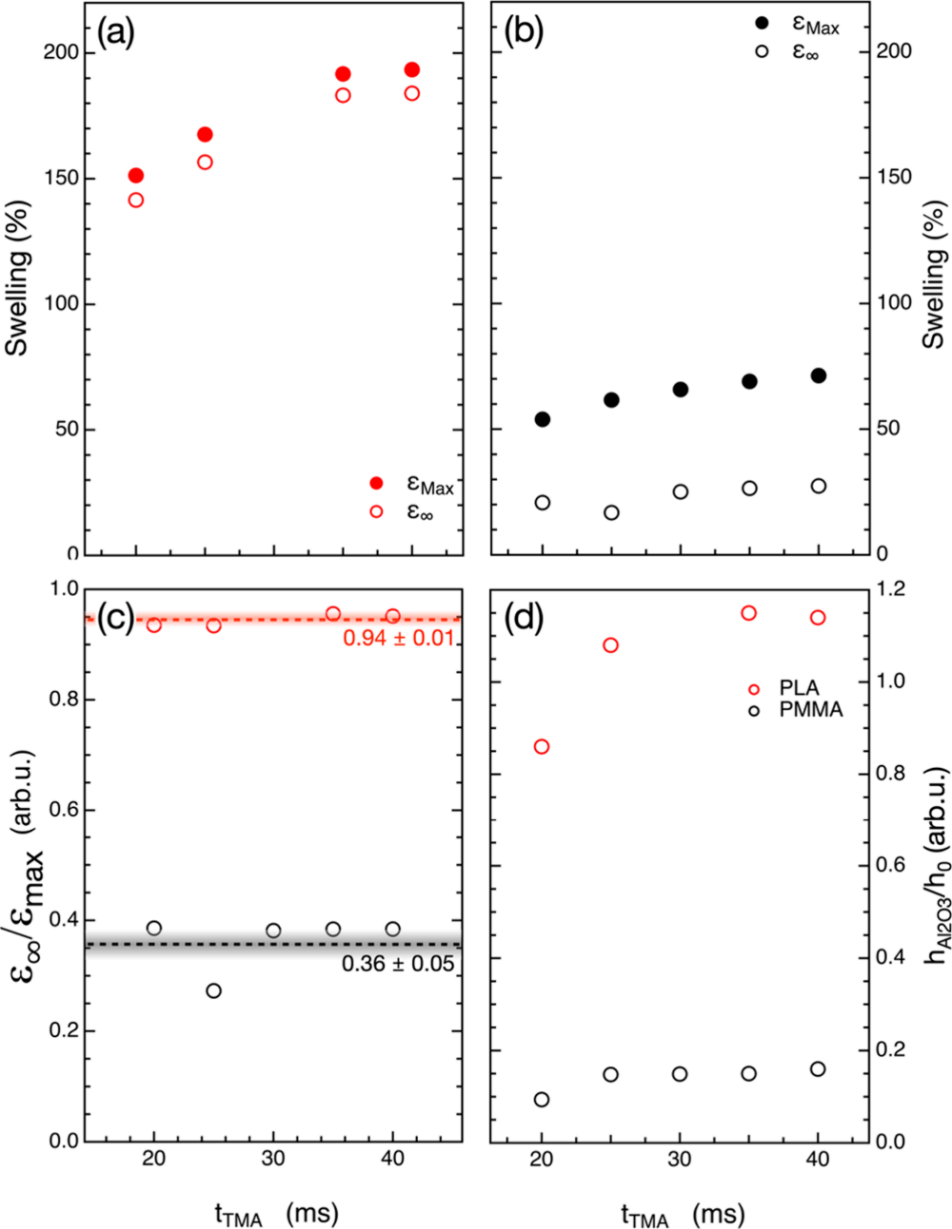
ε_MAX_ (closed symbol) and ε_∞_ (open symbol) for PLA (a) and PMMA (b) reported as a function of TMA exposure time. (c) The ratio ε_∞_/ε_MAX_ is reported as a function of TMA exposure time for PLA (red open symbols) and PMMA (black open symbols), respectively. Dashed lines correspond to the average ε_∞_/ε_MAX_ values for PLA (red) and PMMA (black). (d) Thicknesses of the residual Al_2_O_3_ films upon removal of the PLA (red open symbols) and PMMA (black open symbols) matrices by the O_2_ plasma reported as a function of TMA exposure time. Values are normalized on the thickness, h_0_, of the pristine polymer films.

Nevertheless, the thicknesses of the PLA and PMMA films exhibit no significant increase when heated at *T* = 70 °C during thermalization in the ALD reactor before the SIS process. Accordingly, no significant variation of the free volume occurred in PLA when heated at 70 °C. Consequently, the huge TMA sorption of PLA cannot be explained assuming a large free volume in the polymer matrix because of the processing temperature above *T*_G_. Conversely, we speculate that this huge TMA sorption is related to the specific interaction of TMA with PLA: TMA acts as a plasticizer that significantly reduces the glass transition temperature of the polymer, inducing a significant swelling that promotes further TMA diffusion into the PLA template. Moreover, in a recent paper,^34^ we demonstrated that increasing the processing temperature from 70 to 110 °C, the swelling is progressively reduced in PMMA, due to a progressive reduction of TMA solubility in PMMA. Similar evidence was reported by Petit et al. in a previous paper, when comparing the swelling behavior of PMMA at 75 and 125 °C.^32^ Data in the literature clearly demonstrate that swelling of PMMA during TMA exposure does not significantly increase when performing the infiltration process at temperatures above *T*_G_, suggesting that solubility of TMA into a polymer cannot be simply inferred on the basis of general consideration about the physicochemical structure of the polymer.

During the TMA purging step, the two polymer matrices exhibit significantly different behavior. In particular, PMMA films are characterized by a fast and massive deswelling, fully consistent with previous results in the literature.^27,32^ Conversely, almost no deswelling is observed in the PLA films. Polymer deswelling during the TMA purging step is associated with the desorption of TMA molecules that were not incorporated into the polymer matrix by an irreversible reaction with the reactive sites of the polymer matrix. Information about the fraction of TMA molecules chemically trapped into the polymer matrix is obtained by fitting the deswelling curve with an exponential decay function:

$$\varepsilon (t)=\varepsilon_{\infty }+e^{-t/\tau }$$ 3

where ε_*∞*_ corresponds to the film thickness at an infinite purging time and τ indicates the characteristic time of the deswelling process. The ε_*∞*_ values as a function of *t*_TMA_ are reported in Figure 3a,b for PLA and PMMA, respectively. As highlighted in a very recent paper,^28^ the ratio ε_∞_/ε_MAX_ indicates the fraction of TMA molecules that are stably incorporated into the polymer matrix through a chemical reaction. Interestingly, the ratio ε_∞_/ε_MAX_ is fairly constant (Figure 3c) for both polymers, with average values corresponding to 0.94 ± 0.01 for PLA and 0.36 ± 0.05 for PMMA. These results indicate that almost all of the TMA molecules are adsorbed and chemically trapped into the PLA matrix. Conversely, PMMA exhibits a low TMA incorporation efficiency because of reduced sorption and weaker reactivity with TMA compared to PLA, even if the process is performed at a temperature that is identified to correspond to the thermodynamic equilibrium for the reaction of TMA molecules with the reactive sites of the PMMA matrix.^26^

Further confirmation of this interpretation of the SE data is provided by measuring the thickness of the residual Al_2_O_3_ film that is left over the Si substrate upon removal of the polymer template by a prolonged O_2_ plasma treatment. The thicknesses of the Al_2_O_3_ films upon removal of the PLA and PMMA matrices are reported in Figure 3d as a function of *t*_TMA_. The Al_2_O_3_ thickness values are normalized over the initial thickness *h*_0_ of the polymer films. The normalized thickness values are fairly constant, within the experimental error, for PMMA, irrespective of the *t*_TMA_. In the case of the PLA template, the normalized thickness values of the residual Al_2_O_3_ films progressively increase when increasing *t*_TMA_ and achieves a sort of saturation value for *t*_TMA_ ≥ 35 ms. Additionally, the normalized thickness values of the residual Al_2_O_3_ films upon the removal of the PLA are determined to be around 1 order of magnitude higher than those obtained using PMMA as a template, confirming that the amount of TMA stably incorporated in the PLA films is significantly larger than in the PMMA films, in good agreement with operando SE results.

### X-ray Photoelectron Spectroscopy

As previously discussed, the almost negligible deswelling observed in the case of the PLA films during the TMA purging step points to stable incorporation of TMA molecules into the polymer template, resulting in efficient Al_2_O_3_ infiltration. On the contrary, significant deswelling in PMMA films is clear evidence of the limited reactivity of TMA molecules with the PMMA polymer. Accordingly, ex-situ XPS measurements were performed in order to better clarify this point and identify the specific reactive sites involved in the reaction of TMA with PLA and PMMA polymers.

The low-resolution XPS spectra of pristine and infiltrated PLA and PMMA films are reported in Figure S3. The spectra of the infiltrated samples were acquired by measuring polymer films that were not exposed to X-ray before infiltration in order to rule out any effect related to X-ray-induced damage. All these low-resolution spectra were acquired keeping constant the acquisition time, which was fixed to be 11 min. Accordingly, assuming a constant photon flux from the X-ray source, the spectra can be directly compared without any further normalization. The analysis was restricted to the samples infiltrated using TMA pulse times of 20 and 40 ms. The spectra of the pristine polymer films are characterized by the presence of two main signals at approximately ∼285 and ∼530 eV that correspond to C *1s* and O *1s* core levels, consistently with the chemical structures of the two polymers. The spectra of the infiltrated samples are characterized by two additional peaks at approximately 120 and 75 eV, corresponding to Al *2s* and Al *2p* core levels, respectively. These signals confirm effective incorporation of Al_2_O_3_ into the polymers upon infiltration.^53^ Moreover, the intensity of the Al related signals in the infiltrated PLA films is significantly higher than that in the PMMA samples, indicating that PLA films incorporated more Al_2_O_3_ than the PMMA ones. Quantitative information about the effective Al_2_O_3_ infiltration into the different polymer matrix was obtained by proper analysis of the high-resolution XPS spectra of these samples.

Figure 4a,b reports the high-resolution XPS spectra (open symbols) of the C *1s* and O *1s* signals obtained from pristine and infiltrated PLA films, respectively. The collected spectra show no evidence of evolution as a function of time, demonstrating that the duration of the X-ray exposure is not enough to determine any substantial degradation of the polymer matrix. The C *1s* spectrum of the pristine PLA film is distinctly characterized by the presence of three main components in perfect agreement with data in the literature.^40^ The three components correlate well with the chemical structure of the PLA monomer, as highlighted by the colored semitransparent circles in the inset of Figure 4a. These components are labeled as C=OOC, C–OCCH, and C–CHHH to account for the nearest neighbors of the specific carbon atom associated with each component. The spectrum of the pristine sample was deconvoluted by fitting experimental data with Voight functions (colored area) in order to determine the binding energy of the different components. The fitting parameters for the different components of the pristine sample are reported in Table S1. Similarly, the O *1s* high-resolution spectrum of the pristine PLA film was deconvoluted by fitting the experimental data with two Voight functions (colored solid lines) corresponding to the different chemical configurations of the oxygen atoms in the PLA monomers, as highlighted by colored semitransparent circles in the inset of Figure 4b. Accordingly, these two components have been labeled as O–CC and O=C to account for the nearest neighbors of the specific oxygen atom associated with each component.

**Figure 4:**
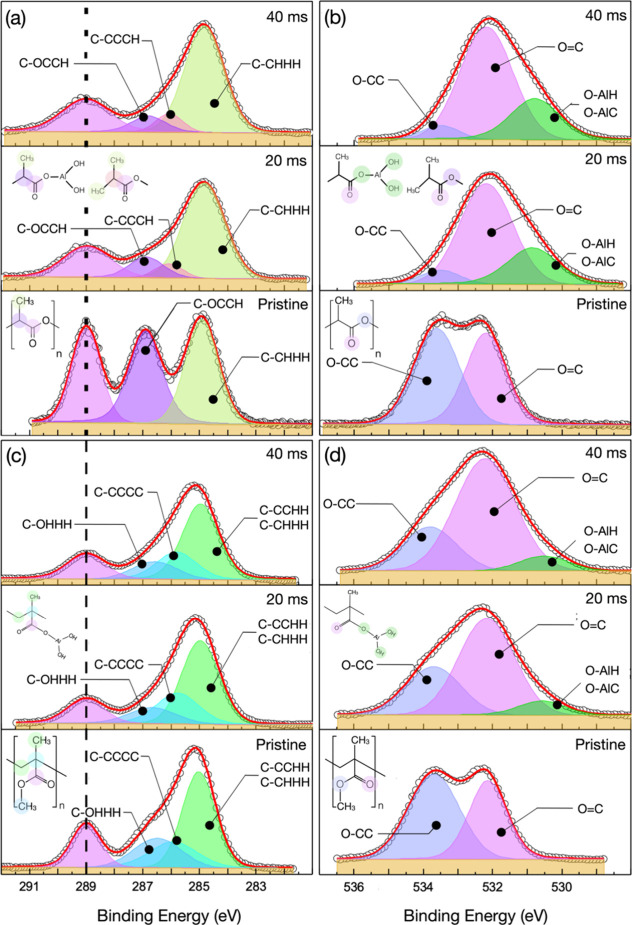
High-resolution XPS spectra (open symbols) of C 1 (a) and O 1s (b) core levels for pristine and infiltrated 15 nm thick PLA films and of C 1 (c) and O 1s (d) core levels for pristine and infiltrated 15 nm thick PMMA films. Black dashed line indicates the binding energy (289.0 eV) of the C 1s core level originated by the carbonyl group that was used as a reference to correct the energy shift of the binding energy induced by charging. Red lines correspond to the best fit of the experimental data resulting from the convolution of the different components. In the insets, the chemical structure of the pristine PLA and PMMA molecules and of the chemical structure of these molecules upon reaction with TMA and H_2_O, with the indication (colored shadows) of the C and O atoms generating the photoelectrons corresponding to the different components of the spectra.

The C *1s* and O *1s* spectra of the infiltrated PLA films are significantly modified compared with those of the pristine sample. In particular, the intensities of the C=OOC and C–OCCH components of the C *1s* spectra are much lower than the intensity of the C–CHHH component, suggesting that the ester group is significantly affected by the interaction with the TMA precursors during the infiltration process. Moreover, an additional component was introduced to properly fit the experimental data. Considering the shift of the binding energy with respect to the C–CHHH component, this new component was identified as C–CCCH. The fitting parameters for the different components in the infiltrated samples are reported in Table S1. Similarly, the O *1s* spectra of the infiltrated samples exhibit a significant reduction of the O–CC component with respect to the O=C component. An additional component of O-AlH at low binding energies was introduced in the fitting of the experimental data and associated with the presence of hydroxy groups bonded to Al atoms. An O–AlC component is expected to be present because of the reaction of the O atoms of the ester group with Al of the TMA precursor. This component is assumed to have a binding energy similar to that of the O-AlH component. The experimental data indicate that the infiltration of Al_2_O_3_ into the PLA matrix takes place through the reaction of TMA with the ester groups that are present in the polymer chains. In particular, Al_2_O_3_ incorporation appears to be mainly associated with the formation of Al–O bonds between the Al atoms of the TMA precursors and the C–O–C oxygen of the ester group, suggesting the occurrence of a polymer chain scission during the TMA reaction as already proposed in the case of TMA reaction with PBS films.^34^

Figure 4c,d shows the high-resolution XPS spectra of the C *1s* and O *1s* signals obtained from pristine and infiltrated PMMA films, respectively. The C *1s* spectrum of the pristine PMMA film is distinctly characterized by the presence of four main components^54^ indicated as C=OCC, C–OHHH, C–CCCC, and C–CCHH, respectively. The last component overlaps with the C–CHHH component that is expected to be present in the C *1s* spectrum of PMMA, according to the chemical structure of the PMMA monomer shown in the inset of Figure 4c. As in the case of PLA, the *1s* spectrum of the pristine PMMA film is deconvoluted in two components corresponding to O–CC and O=C signals. The fitting parameters for the different components of the C *1s* and O *1s* spectra of the pristine PMMA sample are reported in Table S1.

The evolution of the C *1s* and O *1s* spectra of the PMMA films upon infiltration is qualitatively quite similar to the one of PLA. More precisely, the intensities of the C=OOC and C–OHHH components of the C *1s* spectra are much lower than the intensity of the C–CHHH component, suggesting that the ester group is significantly modified because of the interaction with the TMA and H_2_O precursors during the infiltration process, consistent with FTIR data previously reported in the literature.^6^ Interestingly, the C–CCCC component remains fairly constant, indicating that the carbon atom in the polymer backbone is not involved in the reaction with the TMA molecules. The fitting parameters for the different components in the infiltrated samples are reported in Table S1. Similarly, the O *1s* spectra of the infiltrated samples exhibit a significant reduction of the O–CC component with respect to the O=C component. This reduction suggests that the final configuration of the system upon reaction with TMA and H_2_O is characterized by the formation of C–O–Al bonds at the C–O–C oxygens of the ester group. The reduction of the O–CC component is significantly lower than in the case of PLA, suggesting that only a fraction of the C–O–C oxygens in the ester groups is consumed during the SIS process. An additional component O-AlH at low binding energies was introduced to account for Al_2_O_3_ incorporation through the formation of Al–O bonds between the Al atoms of the TMA precursors and the C–O–C oxygens of the ester groups. The low intensity of this component with respect to the one associated with the O=C group further corroborates the idea of a limited Al_2_O_3_ incorporation into the PMMA matrix, in perfect agreement with the results obtained by operando SE measurements.

## Density Functional Theory Results

### Interaction between TMA and PMMA and PLA Polymers

The infiltration chemistry of TMA into PMMA has previously been studied by density functional theory (DFT) using a gas-phase monomer as the model polymer.^26,55^ Our investigated reaction path followed that described for TMA in PMMA from the work of Dandley et al.^55^ In this proposed reaction path, TMA initially forms an adduct with a C=O group in PMMA, yielding a redshift of 65 cm^–1^ in the IR-peak for the C=O stretch and a blueshift of 15–25 cm^–1^ for the C–O IR modes.^55^ From this TMA interaction site, TMA can then decompose by methyl transfer or insertion into the ester bond, yielding further shifts in the C=O and C–O IR-peaks.

In our model system, TMA forms an adduct through an interaction with the C=O group present in PMMA and PLA and these adducts show similar structures, as seen in Figure 5a,c; the full molecular structures are shown in Figure S4. The formation of the adduct is weakly exothermic for both polymers, being −0.44 eV for PLA and −0.59 eV for PMMA. The relatively small interaction energy and the lack of any covalent bond breaking or forming indicate that the interaction of TMA at both polymers can be reversible, with bound TMA potentially being easily released from the polymer through the purge step in an SIS cycle. Upon forming the adduct, the C=O distance lengthens from 1.20 to 1.22 Å in both PLA and PMMA. The TMA molecule adopts a slightly tetrahedral arrangement, with no covalent modifications occurring in either polymer. The most noticeable difference between the adduct structures for both polymers is the distance of the TMA from the backbone of the polymer. TMA is much closer to the polymer backbone in PLA than in PMMA, as the C=O group lies in the PLA backbone compared to its position in a side chain in PMMA.

**Figure 5:**
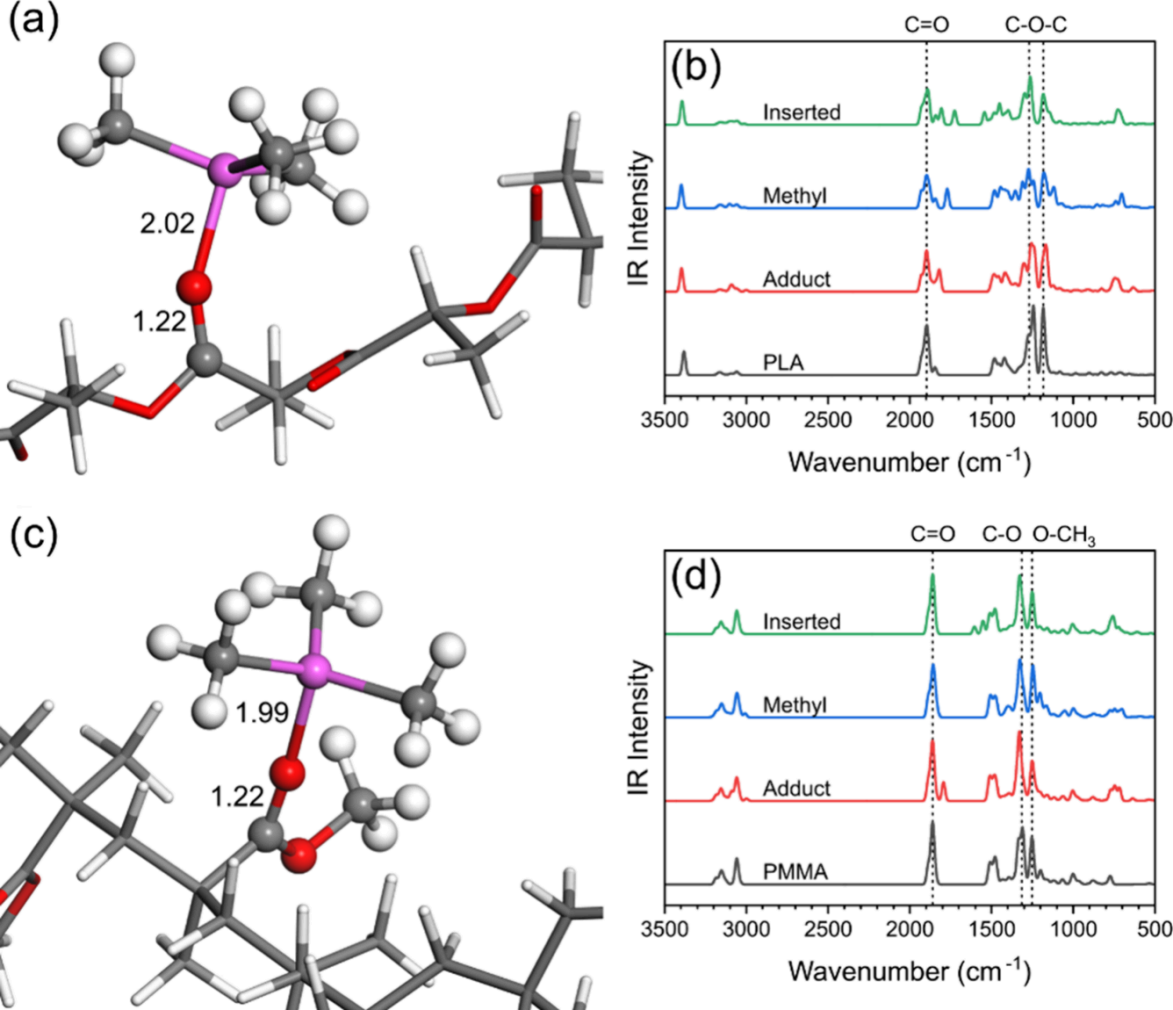
Optimized geometry of TMA forming an adduct with (a) PLA and (c) PMMA. Highlighted bond lengths are in Å. Carbon atoms are gray, hydrogen are white, oxygen are red, and aluminum are magenta. The full geometry is shown in Figure S3. Calculated IR spectra of pristine polymer and with a TMA adduct, methyl-shifted TMA, and inserted TMA for (b) PLA and (d) PMMA.

Figure 5b,d shows the computed IR spectra for the PMMA and PLA oligomers without and with the TMA adduct. The dominant change in the IR spectra after forming the adduct is a redshift of the C=O stretch mode which is 77.45 cm^–1^ for PLA and 66.55 cm^–1^ for PMMA. The redshift value for PMMA agrees well with the previously calculated value of 65 cm^–1^,^55^ and the similar, but larger, magnitude for PLA is consistent with similarities in the TMA-polymer interaction mode and differences in adduct geometry and binding strength.

Further reactions of TMA are needed for it to bind strongly to the polymer, so that it is not released during purging. Two different decomposition pathways for the TMA adduct were then investigated using this nonperiodic model. A methyl transfer product is stable for both PLA and PMMA polymers as seen in Figure 6a,c; the full molecular structures are shown in Figure S5. In PLA, the produced dimethyl aluminum (DMA) coordinates to an oxygen atom in a neighboring C=O in the polymer backbone, while the methyl group has migrated to the carbon center of the initial C=O group. The same coordination cannot occur in PMMA due to neighboring C=O moieties being further away, with the dimethyl only binding to the initial C=O oxygen and the methyl group to the carbon. The double bond in the initial C=O group is reduced to a single bond, as indicated by the increase of its bond length 1.22–1.35 Å for PLA and 1.40 Å for PMMA. For methyl transfer in PLA, the IR peak for the C=O stretch mode of the coordinating carbonyl is redshifted by 125 cm^–1^ indicative of DMA forming a stronger coordination compared to TMA. The redshifted C=O IR peak from the adduct disappears as the bond order is reduced and a new IR peak at 1353.47 cm^–1^ appears corresponding to the new C–O stretch, as shown in Figure 5b,d. The methyl transfer reaction is exothermic for PLA with a reaction energy of −1.44 eV relative to the adduct formation, while the reaction is endothermic for PMMA with a reaction energy of 0.16 eV relative to the adduct. This agrees with previous conclusions that the methyl transfer is not a viable reaction when TMA interacts with PMMA and the TMA:C=O adduct is a more preferred mode of interaction with PMMA.

**Figure 6:**
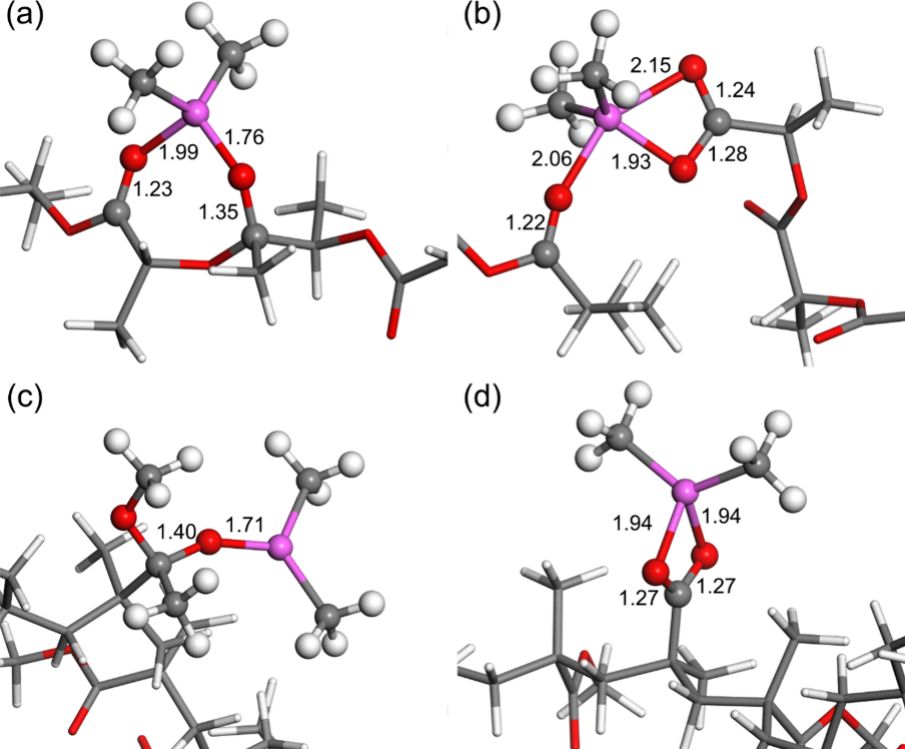
Optimized geometry of the methyl transfer product for PLA (a) and PMMA (c) and the TMA insertion product for PLA (b) and PMMA (d). Highlighted bond lengths in Å. Carbon atoms are gray, hydrogen are white, oxygen are red, and aluminum are magenta. The full geometry is shown in Figures S5 and S6.

The second investigated reaction path was insertion of TMA after ester oxygen. This product was found for both polymers; however, the difference in location of the C=O group between PLA and PMMA strongly influences the final geometry of the product, as shown in Figure 6b,d. When TMA is inserted into PLA, the backbone of the polymer is severed as the DMA chelates to the two oxygen atoms on the carboxyl terminal, while a methyl group is transferred to the carbon-head of the fragment chain. The chain is however able to reattach due to the DMA group cross-linking the two fragments, Figure 6b; the full molecular structures are shown in Figure S6. The insertion reaction is more exothermic than methyl transfer with a reaction energy −2.56 eV relative to the adduct, while breaking the cross-link between the two fragments is endothermic with an energy cost of 0.62 eV. The most notable change in the IR spectrum for TMA insertion into PLA is a redshift of 89.66 cm^–1^ for the C=O stretch mode of the cross-linked oxygen and the addition of two new peaks at 1725.42 and 1542.98 cm^–1^ corresponding to asymmetrical and symmetrical stretch modes of the chelating carboxyl terminal.

For PMMA, the polymer backbone remains intact after TMA insertion, due to the location of the C=O moiety of the side chain. However, an ethane molecule is released from the polymer upon insertion, Figure 6d. This reaction is also exothermic, albeit less so than that for PLA, with a reaction energy of −1.82 eV relative to the adduct. The peak corresponding to the redshifted C=O stretch disappears from the IR spectra upon insertion, and two peaks, corresponding to the asymmetrical and symmetrical stretch modes of the chelating carboxyl group, appear at 1604.34 and 1152.11 cm^–1^, respectively.

Figure 7 shows a scheme summarizing the reaction pathways of TMA with PMMA and PLA. In both cases, the formation of an adduct between the TMA molecule and the C=O moiety is found to be a weakly exothermic reaction that is expected to be reversible at room temperature. Two possible reactions paths are investigated with a methyl transfer between TMA and the polymer chain or the insertion of the TMA molecule into the polymer chain by reaction with the ester oxygen. The methyl transfer reaction is determined to be endothermic for PMMA and exothermic for PLA, indicating this is not a viable path for TMA reaction with PMMA. The more favorable reaction energy for TMA insertion into PLA compared to PMMA is consistent with the experimental XPS, with a stronger reduction in the C–O intensity for PLA compared with PMMA.

**Figure 7:**
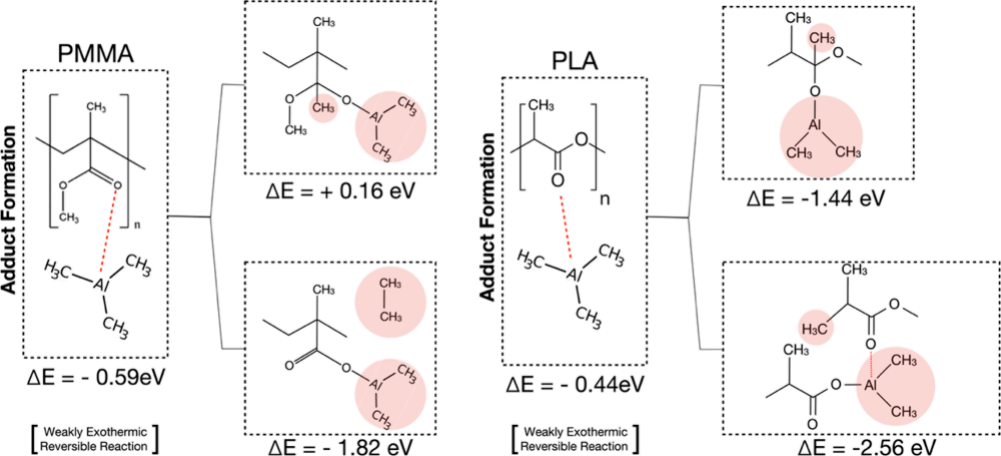
Scheme showing the reaction path of PMMA and PLA with a TMA molecule. Irrespective of the final reaction products, the reaction path goes through the formation of an adduct between the carboxylic group and the TMA molecule via a weak exothermic reaction.

### Swelling of TMA-Infiltrated Polymers

The atomic structure of the polymers together with the volume and the shape of the computational cell were optimized without any TMA introduced, and the calculated density of the polymers is 1.180 g cm^–3^ for PLA, close to the experimental densities of 1.25 and 1.232 g cm^–3^ for PMMA, compared to the experimental density of 1.17–1.20 g cm^–3^. The close agreement of the DFT-calculated density with the experimental density, with a deviation of ca. 5%, indicates that the model can be used to estimate the swelling upon infiltration of TMA.

TMA infiltration was modeled by adding an increasing number of TMA molecules into the polymer supercell, up to a maximum of one TMA per monomer (16 TMA molecules) and optimizing the structure and volume. The TMA molecules were initially placed in close proximity to the C=O groups in the polymers to allow formation of the adduct structure found in the nonperiodic model. The infiltration energy, optimized volume of the cell, and computed swelling are given in Table S2, and the optimized geometry for the polymers with 2 and 16 TMA infiltrated is shown in Figure 8; geometries with 2, 8, 12, and 16 infiltrated TMA are shown in Figure S7.

**Figure 8:**
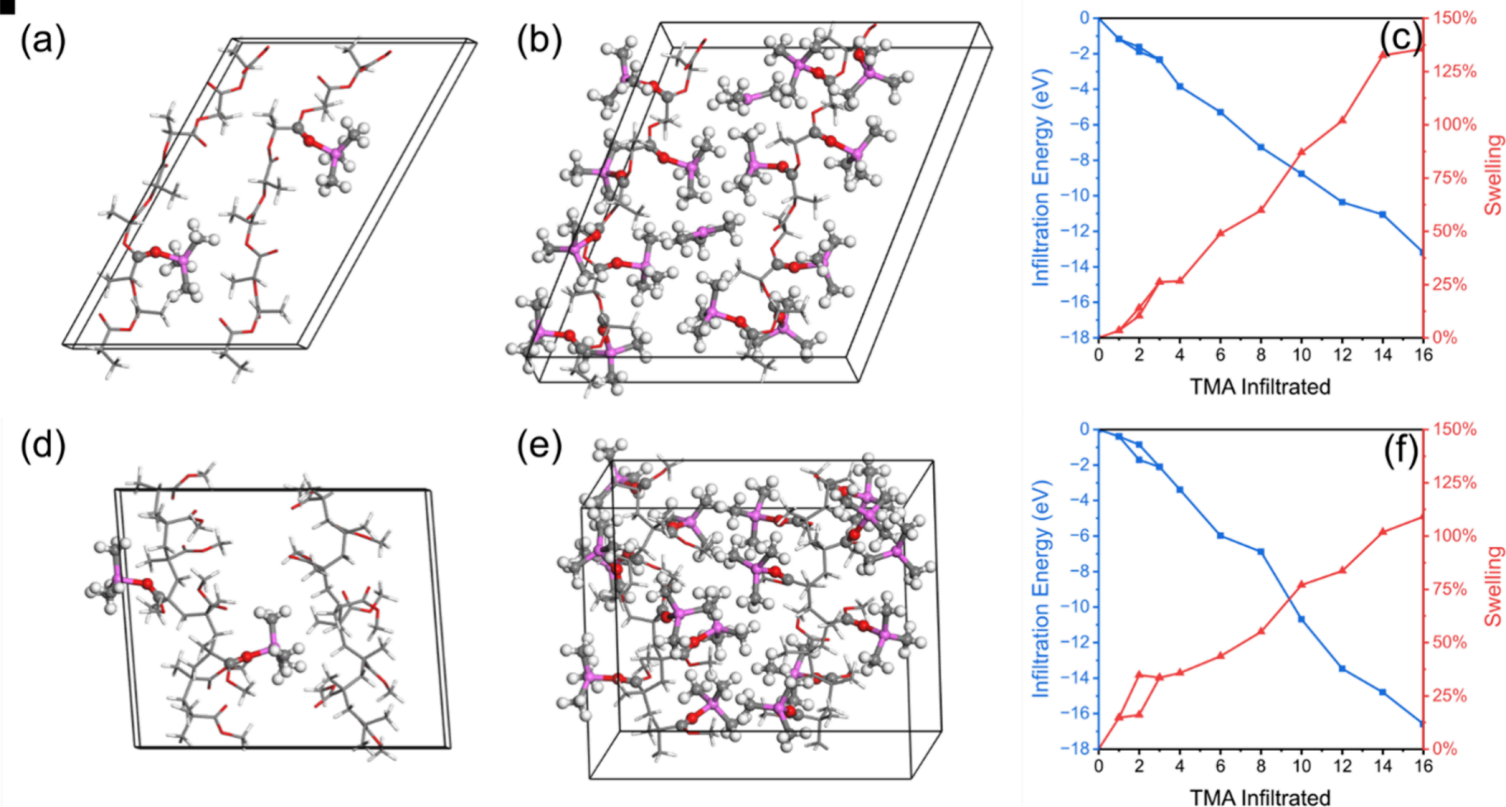
Optimized geometry of PLA (a,b) and PMMA (d,e) with 2 and 16 infiltrated TMA molecules. Carbon atoms are gray, hydrogen are white, oxygen are red, and aluminum are magenta. Infiltration energy (blue) and polymer swelling (red) for an increasing amount of infiltrated TMA (measured by the number of TMA molecules added) into PLA (c) and PMMA (f). Two results are shown for 2 TMA as described in the text.

For two TMA molecules, infiltration was investigated at two different positions in the supercell. In one model, the two TMA molecules are placed close to each other at the same polymer chain, and the other model has the two TMA molecules placed far apart at different polymer chains. The infiltration energy for the TMA molecules follows a generally linear relation for both polymers, as seen in Figure 8c,f, indicating that the binding energy for the TMA to the polymer is independent of saturation level. The average binding energies were calculated to be −0.84 eV for PLA and −1.03 eV for PMMA. The slightly stronger adduct formation energy for PMMA compared to PLA agrees well with the nonperiodic model.

Although both polymers showed swelling upon infiltration of TMA, they showed different behavior. Swelling in PLA followed a generally linear trend starting from 1 TMA, with an average swelling of 8.6% per TMA and reaching a maximum swelling of 136% at saturation. For PMMA, the swelling for the initial two TMA infiltrations was much higher on average 16.8%. Upon addition of more TMA, the swelling was lower, leading to an average swelling of 7.3%/TMA and reaching a maximum swelling of 109% at saturation. This difference in swelling between PMMA and PLA from our DFT model is consistent with the relative swelling observed experimentally by SE analysis.

The linear swelling and initially lower magnitude of swelling observed in PLA can stem from the polymer chain being flexible, and it can therefore distort its structure to accommodate the introduced TMA molecules. The PMMA chains are more rigid, and instead of distorting around the TMA, they initially separate to accommodate the adduct. The two different infiltration positions of two TMA in PMMA are consistent with a more rigid polymer. When an additional TMA molecule is infiltrated close to a TMA adduct, the already created void allows for the second TMA molecule to fit, and only a small swelling is observed. In contrast, if the second TMA molecule is infiltrated far from the original TMA-polymer adduct, then the structure must swell to the same degree as the initial infiltration to fit the additional TMA molecule. This explains the large initial swelling and the subsequently lower swelling afterward: the first TMA molecules separate the PMMA chains creating voids that the following TMA molecules can fit into.

Phonon (vibrational) density of states (VDOS) of pristine and TMA infiltrated PLA and PMMA were calculated using a smaller six monomer, single chain periodic mode. The smaller model gave similar results for swelling and infiltration energy compared to the larger model; for details, see Table S2 in the Supporting Information. The VDOS shows a redshift of the C=O stretch mode corresponding to the adduct formation, as highlighted in Figure 9, consistent with the experimentally observed redshift in the same vibrational mode. The location of the shifted and unshifted peaks is mostly independent of the amount of infiltrated TMA, except for small shifts due to minor structural changes upon adduct formation, and the main difference in the PDOS upon TMA infiltration is the relative size of the two peaks. The average redshift is 45.40 cm^–1^ for PLA and 70.26 cm^–1^ for PMMA, consistent with other results for the shift in this vibrational mode upon adduct formation.

**Figure 9:**
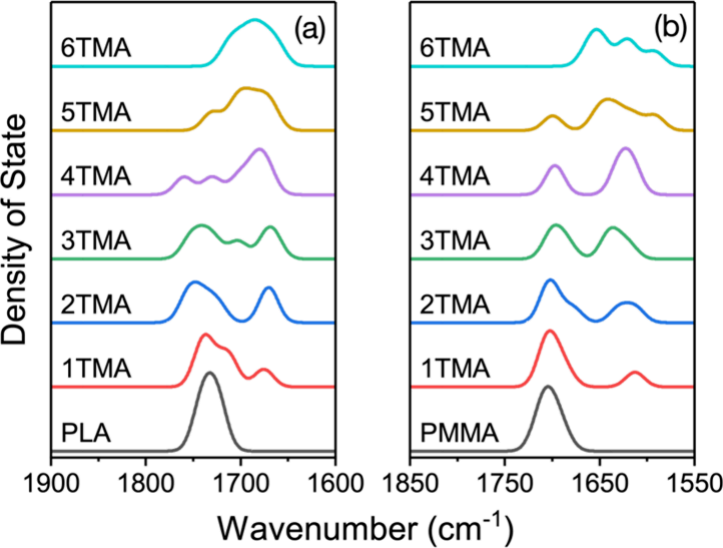
Section of phonon density of states (PDOS) for PLA (a) and PMMA (b) with an increasing number of infiltrated TMA molecules, highlighting the C=O stretch mode. Full PDOS is given in Figure S8 in the Supporting Information.

The six-monomer model was also used to investigate the TMA insertion reaction in a periodic model. Insertion of one, two, and three TMA molecules, corresponding to insertion into half of the monomers, was modeled. Optimized geometries for the insertion product of three TMA molecules are shown in Figure 10. Similar to the nonperiodic model, the PLA chain was severed upon TMA insertion and reattached via the DMA. The placement of the DMA product along the polymer backbone would allow further TMA molecules to diffuse through the polymer to find other binding sites. For PMMA, the location of DMA on the side chain allowed higher TMA mobility, and TMA can form adducts with adjacent C=O groups, both on the same chain and on neighboring chains. This would form a cross-linked network of polymer chains that both blocks binding sites for additional TMA and hinders diffusion of TMA into the polymer, so that the TMA-C=O adduct would dominate. This yields a smaller swelling in PMMA and the observed decrease in swelling seen experimentally upon purging.

**Figure 10:**
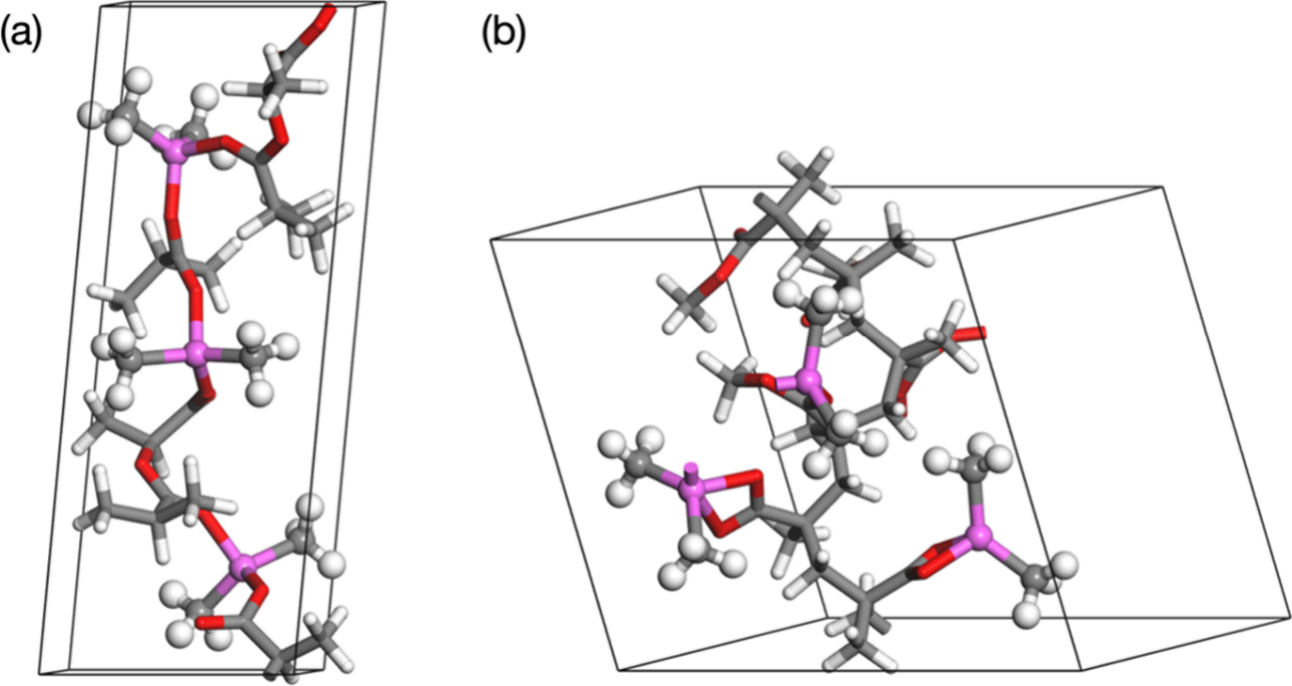
Optimized geometry for the insertion product of three TMA molecules for PLA (a) and PMMA (b). Two of the DMA fragments cross-link with other C=O groups over the periodic boundary for PMMA. Carbon atoms are gray, hydrogen are white, oxygen are red, and aluminum are magenta.

## Conclusions

In this work, Al_2_O_3_ was infiltrated in PMMA and PLA thin films with an ALD reactor, operating at 70 °C in quasi-static mode, using TMA and H_2_O. These polymers are characterized by the presence of ester groups along the polymer chain that are expected to act as reactive sites for TMA. Operando spectroscopic ellipsometry revealed that significant swelling and deswelling of PMMA occur during TMA exposure and purging, respectively, due to sorption and subsequent desorption of TMA molecules that are not stably incorporated into the PMMA matrix. PLA exhibited a much larger swelling than PMMA during TMA exposure, but no significant deswelling was observed during purging, suggesting that a large amount of infiltrated TMA molecules can be effectively trapped into the polymer matrix by a stable chemical bond. Accordingly, ex-situ XPS analysis demonstrated that much more Al_2_O_3_ is grown in PLA than in PMMA. The ex-situ XPS analysis also shows that in both polymers, TMA incorporation mainly occurs through the formation of an Al–O covalent bond at the C–O–C group, similar to other polymers like PET-G, PCL, and PBS. Two density functional theory (DFT) approaches were used to investigate the infiltration of TMA into PMMA and PLA. Binding configurations, energies, and vibrational spectra were modeled using a gas-phase model of a ten-unit oligomer. For both polymers, TMA forms an adduct with the oxygen in C=O with an exothermic reaction energy, consistent with the experiment. Furthermore, TMA was able to exothermically insert into the C–O–C bond of PLA, forming a covalent Al–O bond, aligning with the ex-situ XPS results. Infiltration modeling employed a periodic model from which we show that PMMA and PLA swell upon TMA infiltration, saturating with increasing TMA, consistent with experimental findings. This combined experimental and theoretical study provides deeper insights into SIS of Al_2_O_3_ in PMMA and PLA. This methodology can be extended to other precursors and polymer pairs, allowing the unravelling of the complexities of SIS at the molecular level.

## Supplementary Material



## Notes

Fitting parameters of the high-resolution XPS spectra, infiltration energy, volume, full images of nonperiodic geometries, swelling and lattice constants for infiltrated PLA and PMMA, geometries for 2, 4, 8, 12, and 16 infiltrated TMA, and phonon density of states for infiltrated PLA and PMMA (PDF)
