# Supplementary material for: On the Differences in Trimethylaluminum Infiltration into PMMA and PLA Polymers for Sequential Infiltration Synthesis: Insights from Experiments and First-Principles Simulations
Source: ACS Appl Polym Mater. 2025 Sep 15;7(18):12707–19. doi: 10.1021/acsapm.5c02680 (PMC12481621; doi:10.1021/acsapm.5c02680)
Supplement: Supplementary file 1 [file ap5c02680_si_001.pdf]

Supporting Information

On the differences in trimethylaluminum infiltration into PMMA and PLA polymers for sequential infiltration synthesis: insights from experiments and first principles simulations

Michele Perego[1,], Alessia Motta[1,2§], Karl Rönnby[3§], Forest Tung Jie Yap[3], Gabriele Seguini[1], Claudia Wiemer[1], Michael Nolan[3,*]*

[1] CNR-IMM, Unit of Agrate Brianza, Via C. Olivetti 2, I-20864 Agrate Brianza, Italy

[2] Politecnico di Milano, Dipartimento di Energia, Via Ponzio 34/3, 20133 Milano, Italy

[3] Tyndall National Institute, Lee Maltings, University College Cork, T12R5CP, Ireland

[*] Corresponding authors: michele.perego@cnr.it , michael.nolan@tyndall.ie

[§] these authors contributed equally

Table S1. *Binding energies and FWHMs (in brackets) for the different components of the high resolution C 1s and O 1s spectra in the case of pristine and infiltrated PMMA and PLA thin films. The reported values are assumed to have an average experimental error of ± 0.1 eV. The C=OCC component of the C 1s spectrum has been used as a reference to calibrate the binding energy scale.*

		PMMA			PLA		
		Pristine	Infiltrated		Pristine	Infiltrated	
			20 ms	40 ms		20 ms	40 ms
C *1s*	C=OOC	289.0 eV (1.2 eV)	289.0 eV (1.5 eV)	289.0 eV (1.6 eV)	289.0 eV (1.2 eV)	289.0 eV (1.9 eV)	289.0 eV (2.0 eV)
	C-OCCH	-	-	-	286.9 eV (1.4 eV)	286.9 eV (1.4 eV)	286.9 eV (1.4 eV)
	C-OHHH	286.5 eV (2.0 eV)	286.7 eV (2.0 eV)	286.6 eV (1.9 eV)	-	-	-
	C-CCCC	285.9 eV (2.0 eV)	285.8 eV (1.8 eV)	285.8 eV (1.7 eV)	-	-	-
	C-CCCH	-	-	-	-	286.1 eV (1.1 eV)	286.1 eV (1.1 eV)
	C-CHHH	285.0 eV (1.4 eV)	285.0 eV (1.5 eV)	285.0 eV (1.5 eV)	284.9 eV (1.4 eV)	284.8 eV (1.7 eV)	284.8 eV (1.6 eV)
O *1s*	O=C	533.7 eV (1.3 eV)	533.7 eV (1.9 eV)	533.8 eV (2.0 eV)	533.6 eV (1.5 eV)	533.5 eV (1.4 eV)	533.6 eV (1.4 eV)
	O-CC	532.1 eV (1.3 eV)	532.2 eV (1.9 eV)	532.2 eV (2.0 eV)	532.2 eV (1.4 eV)	532.2 eV (1.8 eV)	532.2 eV (1.8 eV)
	O-AlH	-	530.6 eV (1.6 eV)	530.5 eV (1.7 eV)	-	530.9 eV (1.7 eV)	530.8 eV (1.7 eV)

Table S2. *Calculated infiltration energy, volume, swelling and lattice constants for PLA and PMMA infiltrated by TMA.*

Infiltrated TMA	Infiltration energy (eV)	Volume (Å^3)	Swelling (%)	Lattice constants					
				a (Å)	b (Å)	c (Å)	α (°)	β (°)	γ (°)
PLA									
0	0.00	1622.5	0.0	13.5	23.0	5.7	83.9	92.5	66.0
1	-1.17	1678.9	3.5	13.7	23.0	5.9	84.4	94.4	67.1
2 near	-1.89	1790.4	10.4	15.1	23.7	5.7	89.1	93.3	61.3
2 far	-1.62	1850.5	14.1	15.9	23.6	5.7	90.8	97.0	60.9
3	-2.34	2046.0	26.1	17.5	23.6	5.7	88.9	93.9	60.6
4	-3.84	2055.8	26.7	17.7	23.5	5.7	87.9	92.2	59.6
6	-5.30	2415.2	48.9	17.8	24.0	6.3	83.7	87.6	65.2
8	-7.27	2593.5	59.9	19.8	24.5	6.0	80.0	86.7	65.1
10	-8.76	3034.6	87.0	20.4	23.4	7.6	76.6	88.8	60.6
12	-10.36	3277.5	102.0	21.8	23.5	7.6	78.7	83.9	59.0
14	-11.06	3772.3	132.5	21.3	24.1	8.2	81.8	89.2	65.2
16	-13.20	3823.8	135.7	21.5	24.2	8.1	81.1	96.0	68.2
PMMA									
0	0.00	2159.7	0.0	15.7	17.4	7.9	89.4	87.3	93.6
1	-0.40	2477.2	14.7	18.0	17.5	7.9	89.0	87.3	94.6
2 near	-1.70	2508.2	16.1	18.1	17.6	7.9	88.4	89.1	93.0
2 far	-0.85	2910.7	34.8	21.0	17.5	8.0	89.0	92.7	94.4
3	-2.11	2884.5	33.6	20.8	17.5	7.9	88.5	91.9	92.9
4	-3.39	2930.9	35.7	21.0	17.6	8.0	87.1	92.3	93.1
6	-5.99	3102.4	43.6	21.6	17.5	8.2	86.0	90.1	93.0
8	-6.88	3348.8	55.1	20.9	18.3	9.1	74.8	92.1	93.1
10	-10.69	3823.5	77.0	19.0	18.1	11.9	73.4	78.4	91.3
12	-13.47	3967.5	83.7	19.3	18.2	12.2	71.8	75.8	86.5
14	-14.81	4358.2	101.8	21.1	18.1	12.2	74.8	78.6	91.8
16	-16.58	4516.9	109.1	21.6	18.1	12.2	75.1	80.4	91.8
PLA (small)									
0	0.00	572.3	0.0	7.1	17.6	5.5	82.5	88.6	57.3
1	-0.82	669.7	17.0	8.0	17.0	5.8	75.5	103.2	70.4
2	-1.59	836.8	46.2	8.2	16.3	8.2	71.7	120.0	81.5
3	-2.08	1031.1	80.2	9.6	17.1	7.8	87.1	95.2	50.7
4	-3.12	1166.2	103.8	8.9	17.3	8.7	73.3	108.1	80.2
5	-3.91	1295.2	126.3	9.3	17.8	8.5	82.4	101.5	72.0
6	-5.28	1322.4	131.1	10.2	18.2	8.5	82.4	108.0	66.1
PMMA (small)									
0	0.00	785.1	0.0	7.2	14.8	7.7	99.4	104.1	82.0
1	-1.10	914.4	16.5	9.7	14.0	7.4	91.8	113.5	88.0
2	-1.96	1012.4	29.0	11.1	14.2	7.5	83.5	120.3	89.9
3	-3.29	1136.8	44.8	11.0	14.2	9.1	98.0	125.6	88.0
4	-3.81	1277.0	62.7	11.6	14.5	9.8	87.2	128.7	90.8
5	-4.99	1439.6	83.4	12.4	14.3	10.4	91.1	127.8	95.6
6	-5.72	1550.1	97.4	12.6	14.4	10.3	89.7	122.8	96.8

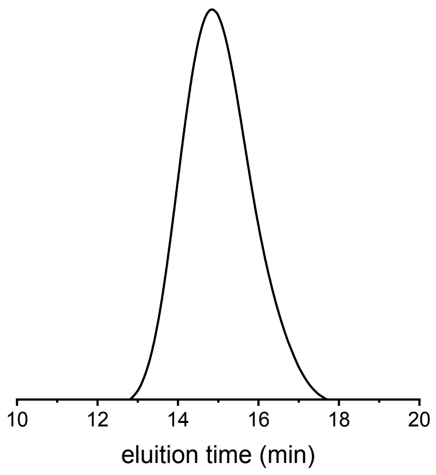

Figure S1. *SEC analysis of a PLA sample. Collected data indicate that the molecular weight of the PLA is* M_n *= 151 kg/mol and the polydispersity index is PDI = 1.24.*

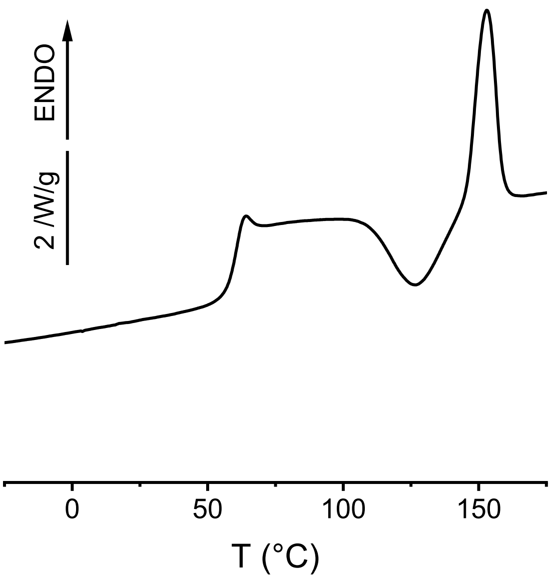

Figure S2. *The PLA sample for DSC analysis was prepared by dissolving the PLA film in chloroform. Then the sample was deposited in the DSC crucible and the solvent evaporated. The DSC thermogram consists of a step due to the glass transition at 63°C (midpoint), an exothermic peak due to a cold crystallization with a minimum at 126°C partly superposed to the endothermic peak at 150°C associated to the melting transition. This indicates that the sample obtained after solvent removal is amorphous or with a degree of crystallinity lower than the maximum attainable value. Unfortunately, the superposition of the cold crystallization exotherm to the melting endotherm makes it impossible to make a correct estimate of the degree of crystallinity eventually present in the sample.*

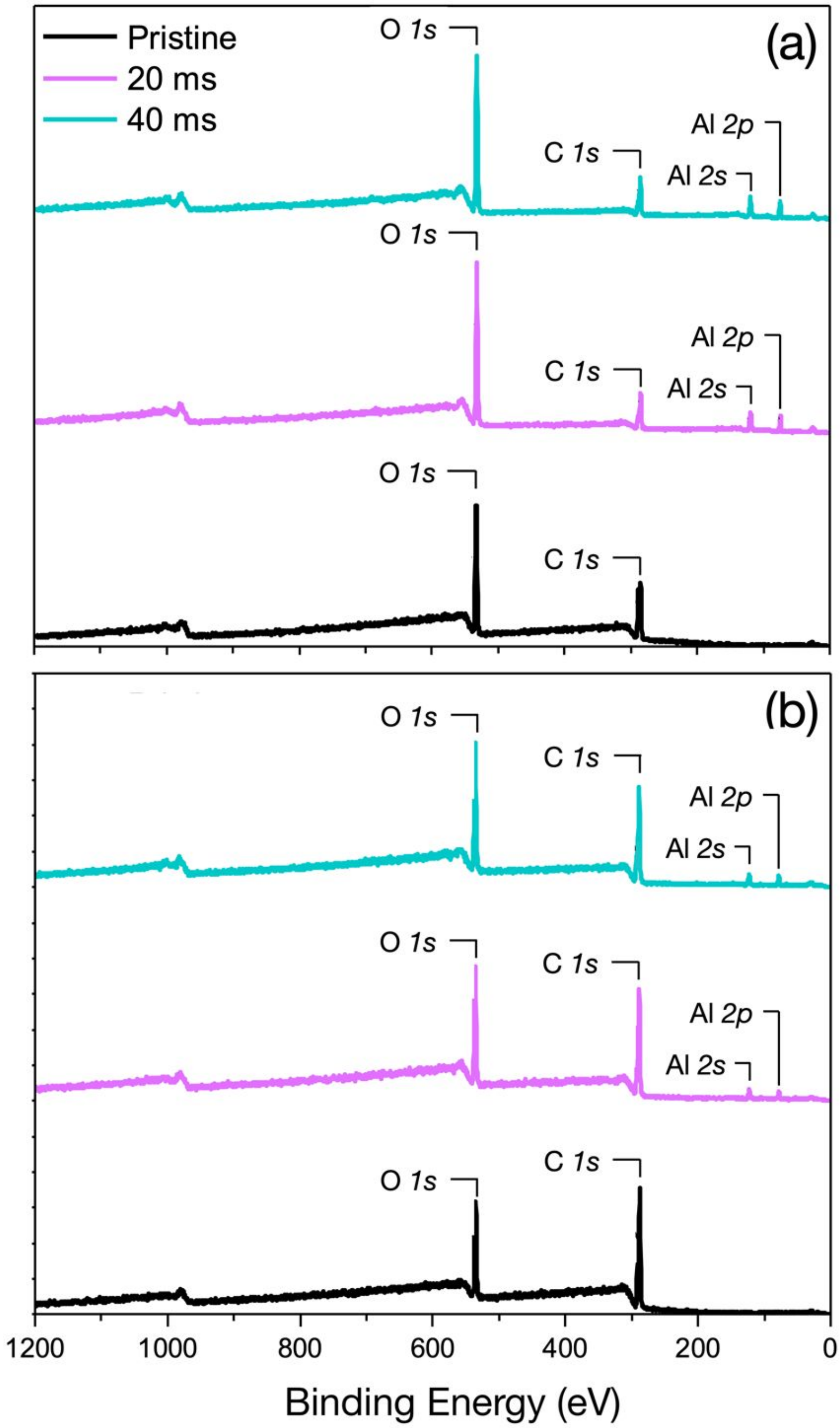

Figure S3. Low resolution XPS spectra of pristine and infiltrated 15 nm thick PLA (a) and PMMA (b) films. Main core level peaks are identified for each spectrum.

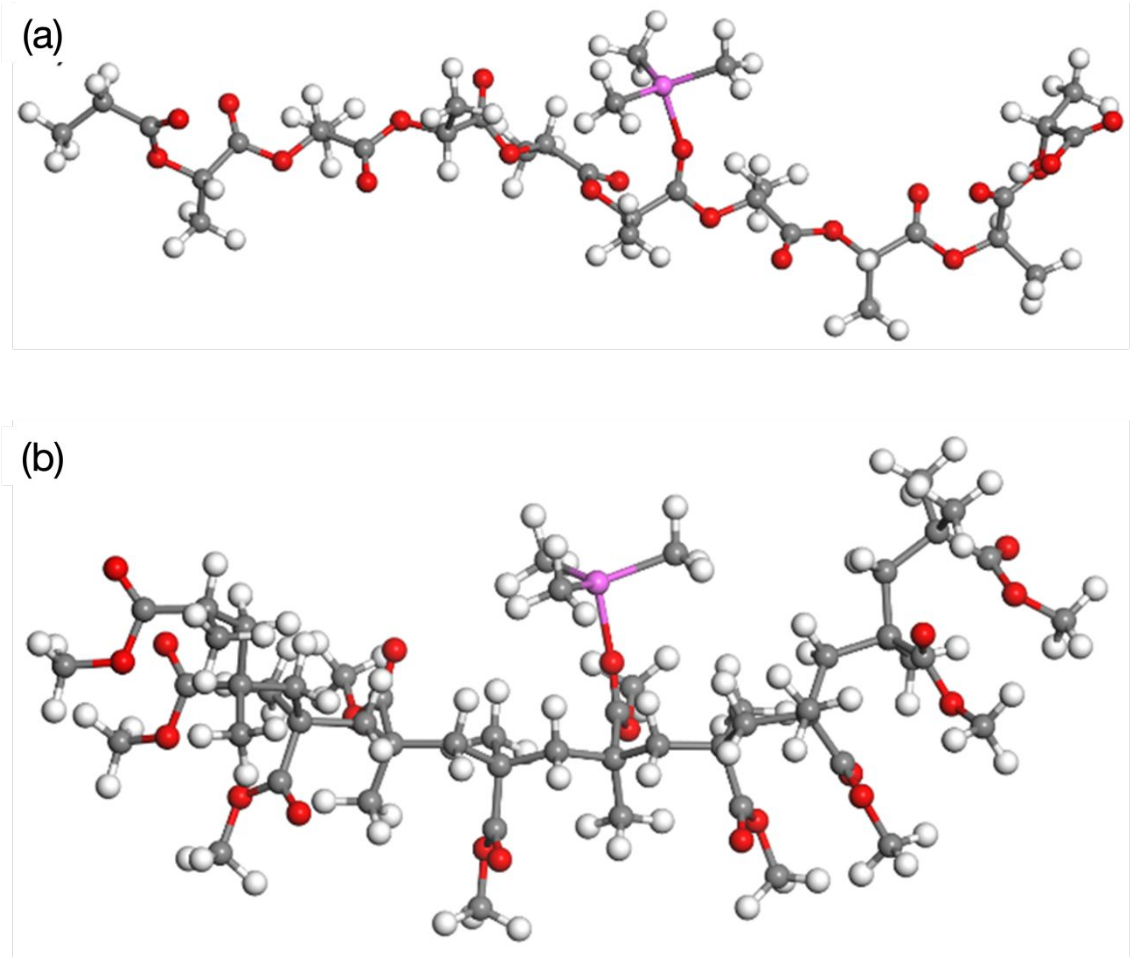

Figure S4. *Optimized geometry of TMA forming an adduct with (a) PLA and (b) PMMA. Carbon atoms are grey, hydrogen are white, oxygen are red, and aluminum are magenta.*

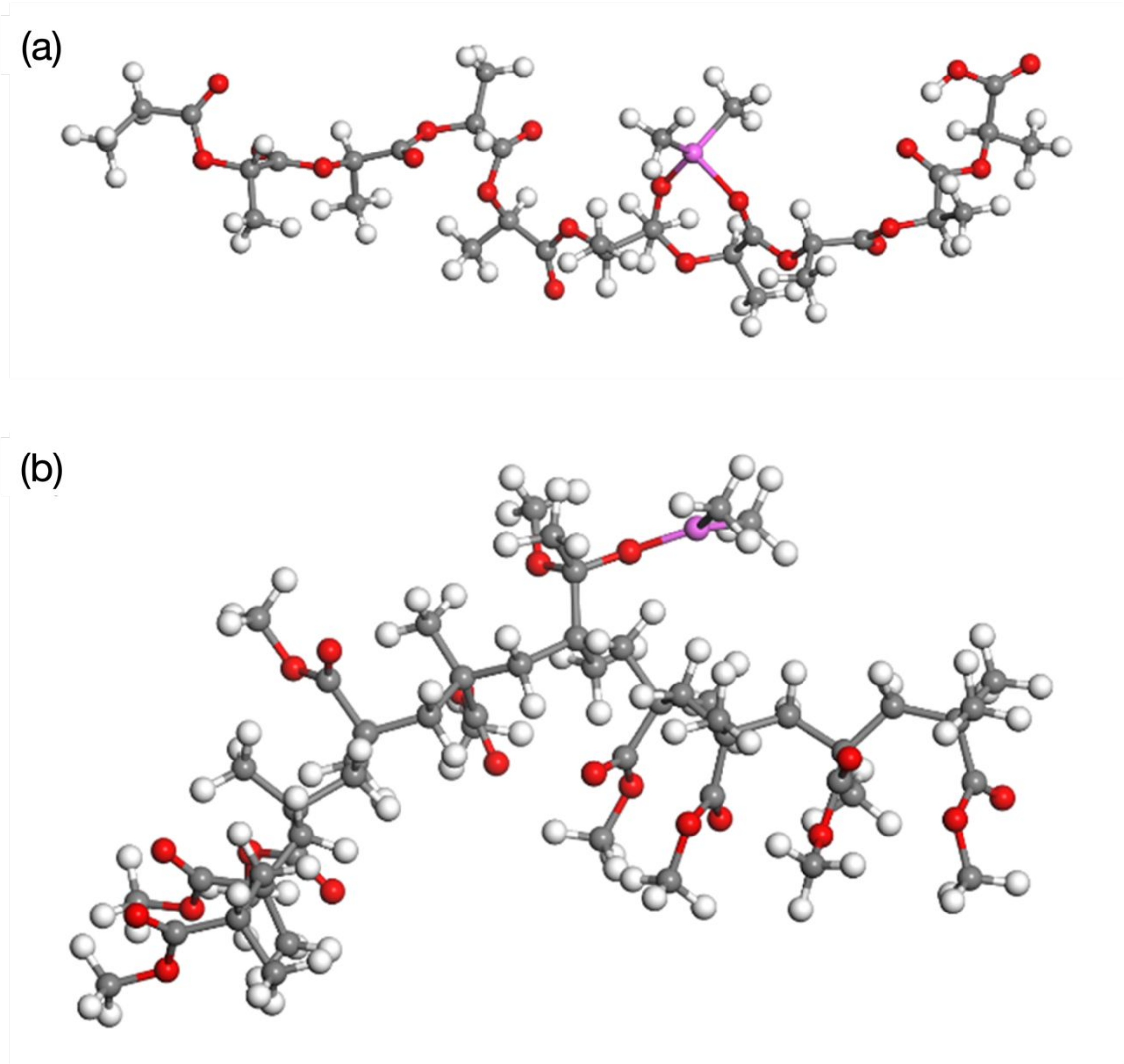

Figure S5. *Optimized geometry of the methyl transfer product for PLA (a) and PMMA (b). Carbon atoms are grey, hydrogen are white, oxygen are red, and aluminum are magenta.*

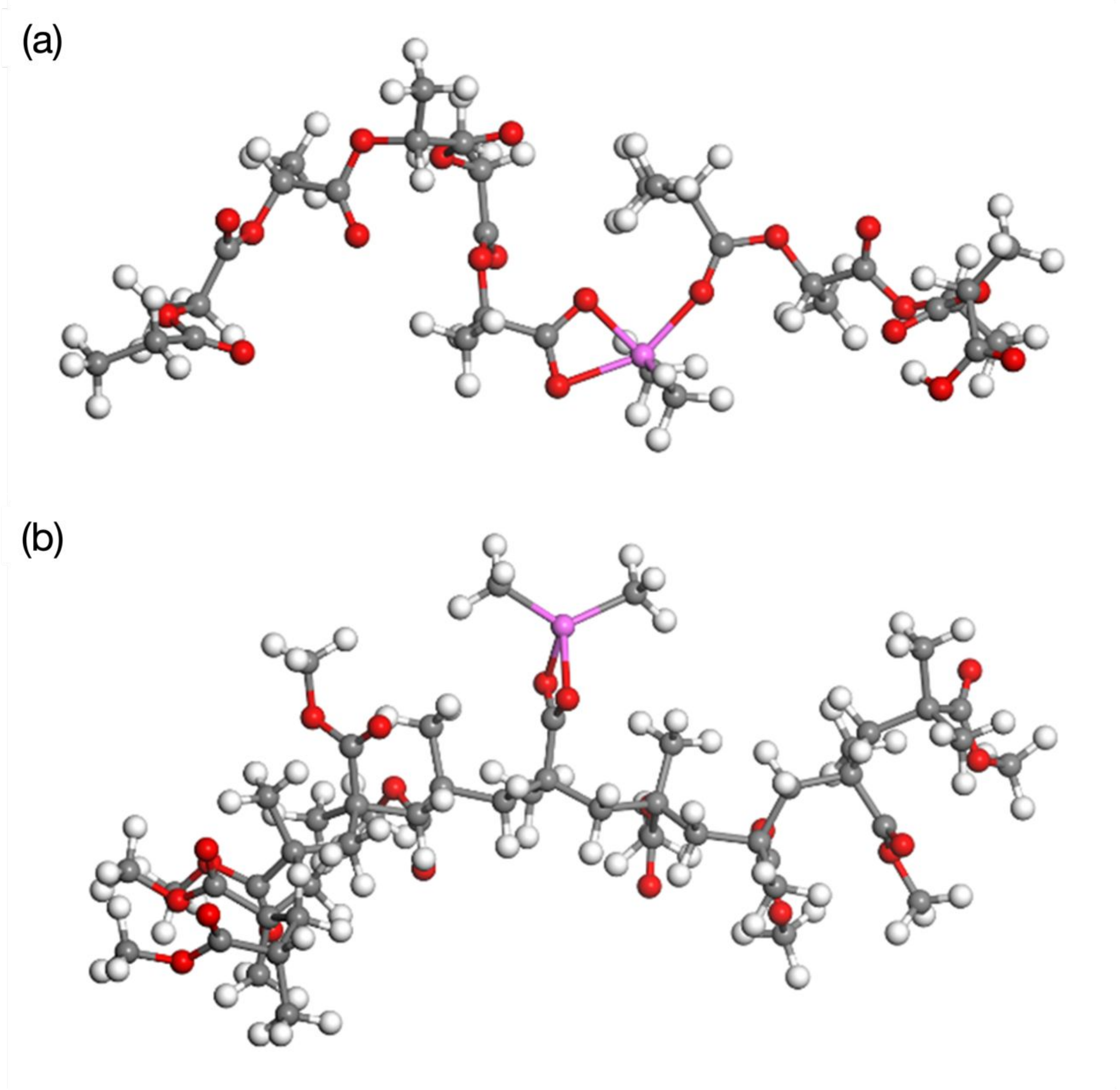

Figure S6. *Optimized geometry of the TMA insertion product for PLA (a) and PMMA (b). Carbon atoms are grey, hydrogen are white, oxygen are red, and aluminum are magenta.*

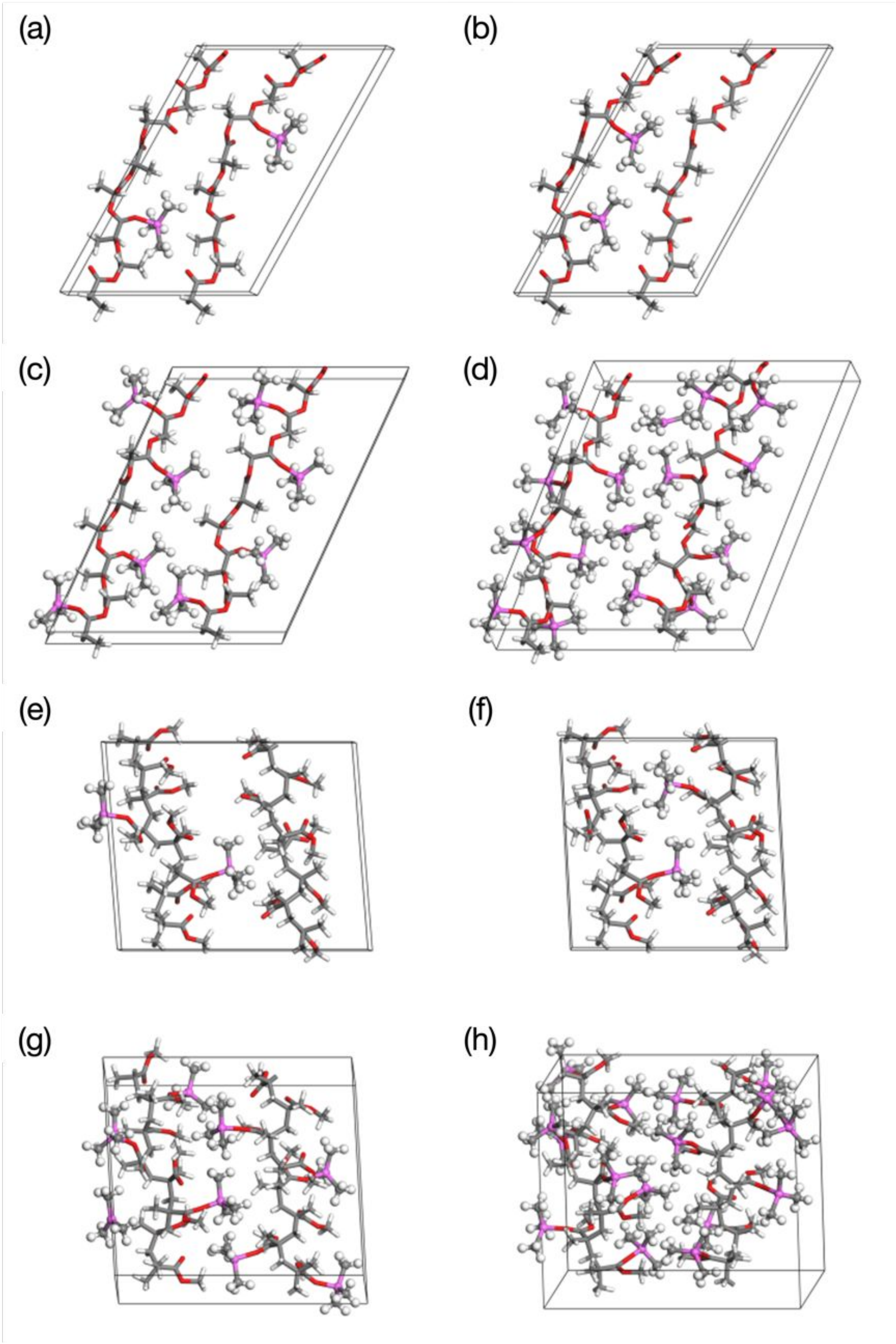

Figure S7. *Optimized geometry of PLA (a-d) and PMMA(e-h) with 2, 8 and 16 infiltrated TMA molecules. The "near" configurations of two TMA molecules are shown in (a) and (e), and the "far" are shown in (b) and (f). Carbon atoms are grey, hydrogen are white, oxygen are red, and aluminum are magenta.*

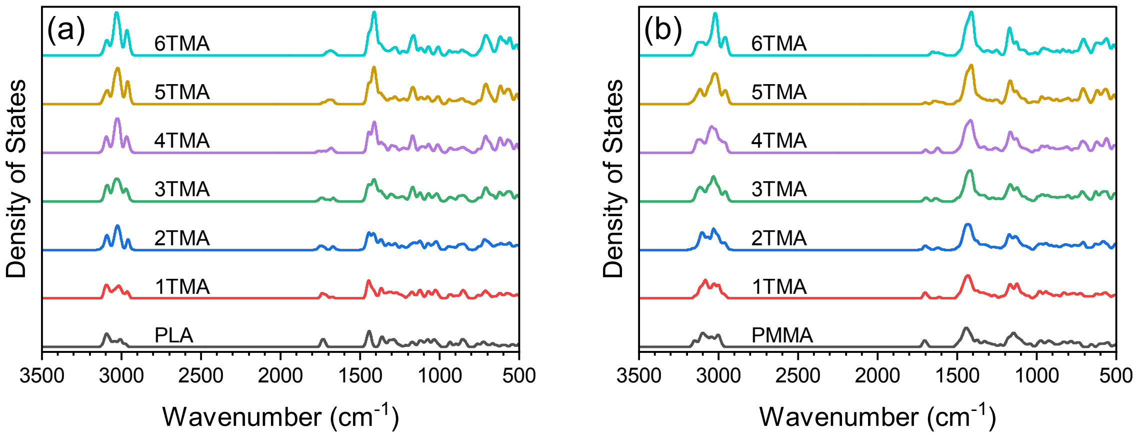

Figure S8. *Phonon density of states for PLA (a) and PMMA (b) with increasing number of infiltrated TMA molecules.*
